# Assessment of common risk factors of diabetes and chronic kidney disease: a Mendelian randomization study

**DOI:** 10.3389/fendo.2023.1265719

**Published:** 2023-09-13

**Authors:** Shuwu Zhao, Yiming Li, Chen Su

**Affiliations:** ^1^ Department of Pain, Hunan Cancer Hospital/The Affiliated Cancer Hospital of Xiangya School of Medicine, Changsha, Hunan, China; ^2^ School of Basic Medicine Science, Naval Medical University/Second Military University, Shanghai, China

**Keywords:** type 1 diabetes, type 2 diabetes, chronic kidney disease, risk factors, Mendelian randomization

## Abstract

**Background:**

The increasing prevalence of diabetes and its significant impact on mortality and morbidity rates worldwide has led to a growing interest in understanding its common risk factors, particularly in relation to chronic kidney disease (CKD). This research article aims to investigate the shared risk factors between type 1 diabetes (T1D), type 2 diabetes (T2D), and CKD using a Mendelian randomization (MR) design.

**Methods:**

The study utilized genome-wide association study (GWAS) datasets for T1D, T2D, and CKD from the FinnGen research project. GWAS summary statistics datasets for 118 exposure traits were obtained from the IEU OpenGWAS database. MR analyses were conducted to examine the causal relationships between exposure traits and each of the three outcomes. Multiple methods, including inverse-variance weighted, weighted median, and MR-Egger, were employed for the MR studies.

**Results:**

Phenome-wide MR analyses revealed that eosinophil percentage exhibited a significant and suggestive causal association with T1D and CKD, respectively, suggesting its potential as a shared risk factor for T1D and CKD. For T2D, 34 traits demonstrated significant associations. Among these 34 traits, 14 were also significantly associated with CKD, indicating the presence of common risk factors between T2D and CKD, primarily related to obesity, height, blood lipids and sex hormone binding globulin, blood pressure, and walking pace.

**Conclusion:**

This research has uncovered the eosinophil percentage as a potential common risk factor for both T1D and CKD, while also identifying several traits, such as obesity and blood lipids, as shared risk factors for T2D and CKD. This study contributes to the understanding of the common risk factors between diabetes and CKD, emphasizing the need for targeted interventions to reduce the risk of these diseases.

## Introduction

The prevalence of diabetes has been raised worldwide and is recognized as a leading cause of high morbidity rates (1). The global number of individuals with diabetes is projected to reach 642 million by 2040, up from 415 million in 2015 (2). Furthermore, diabetes imposes a significant economic burden due to management costs and escalating complications (3). Type 1 diabetes (T1D) is featured by the failure of insulin production due to the destruction of pancreatic β-cells caused by autoimmunity mediated by T-cells (4). In contrast, type 2 diabetes (T2D) is featured by insulin resistance and decreased insulin production (5). Chronic kidney disease (CKD) is a condition related to the progressive malfunction of kidney over time (6), which can occur due to physical injury or conditions such as high blood pressure (7). Patients with CKD face a higher risk of kidney failure compared to individuals with normal kidney function (8). Early detection and treatment can help prevent or delay many complications associated with CKD (9).

Diabetes mellitus (DM) is a major cause of CKD (10). Additionally, diabetes and CKD, share common risk factors like obesity and high blood pressure (11, 12). Extensive research has established the linkage between clinical and lifestyle risk factors and the enhanced risk of noncommunicable diseases like DM and CKD. However, there has been limited investigation into the overlapping risk factors for these main noncommunicable diseases. Mendelian randomization (MR) is an analytical approach where genetic variations serve as instrumental tools (IVs) to represent exposure variables. MR analyses can be used to determine causation and minimize bias resulting from reverse causality and confounding factors (13). In this study, MR analyses were employed to investigate the common risk factors for diabetes and CKD, with the aim of exploring potential strategies for effectively lowering the risks associated with these diseases through focused efforts.

## Methods

All three genome-wide association study (GWAS) datasets for T1D, T2D, and CKD were obtained from the FinnGen research project to enable a better comparison of the common risk factors of these three diseases. For the exposures, this study utilized GWAS summary statistics datasets from the IEU OpenGWAS, which includes a compilation of comprehensive GWAS summary datasets that are accessible as open-source files for downloading or through querying the complete dataset repository. A total of 118 traits were included as exposure variables in the current study, and the selection procedure closely resembled that employed in a recent study by Walker et al. (14). The 118 exposure traits contain variables from various categories, such as anthropometric measurements, biochemistry markers and lifestyle behaviors. **Supplementary Tables 1**-**3** contain the trait information. MR analyses were conducted to examine the causal relationships between exposure traits and each of the three outcomes (T1D, T2D, and CKD). The status of common risk factors of these three diseases was illustrated by a Venn diagram.

In the MR investigations, the selection of IVs for exposures took into account several considerations. Firstly, the selected genetic variations should exhibit a strong and robust association with the exposure, and the P-value threshold was set at P < 5×10^−8^. Secondly, a linkage disequilibrium threshold of R^2^ < 0.001 was applied, along with clumping within a 10-Mb window. The traits with less than 5 IVs in the IV preparation step were excluded from the analyses. For the MR studies, three methods were employed: the inverse-variance weighted (IVW) method, weighted median (WM) method, and MR-Egger. The IVW method was used as primary method in MR analyses because IVW method has the highest effectiveness when all IVs are deemed valid. The MR-Egger intercept test was applied to assess potential horizontal pleiotropy which is a potential limitation of MR analyses because it contradicts the fundamental assumption of MR study. Multiple comparison correction was performed using a 5% false-discovery rate (FDR). The MR studies utilized modified code from a recent publication (14), employing the R package TwoSampleMR for the MR analyses.

## Results

**Figure 1:**
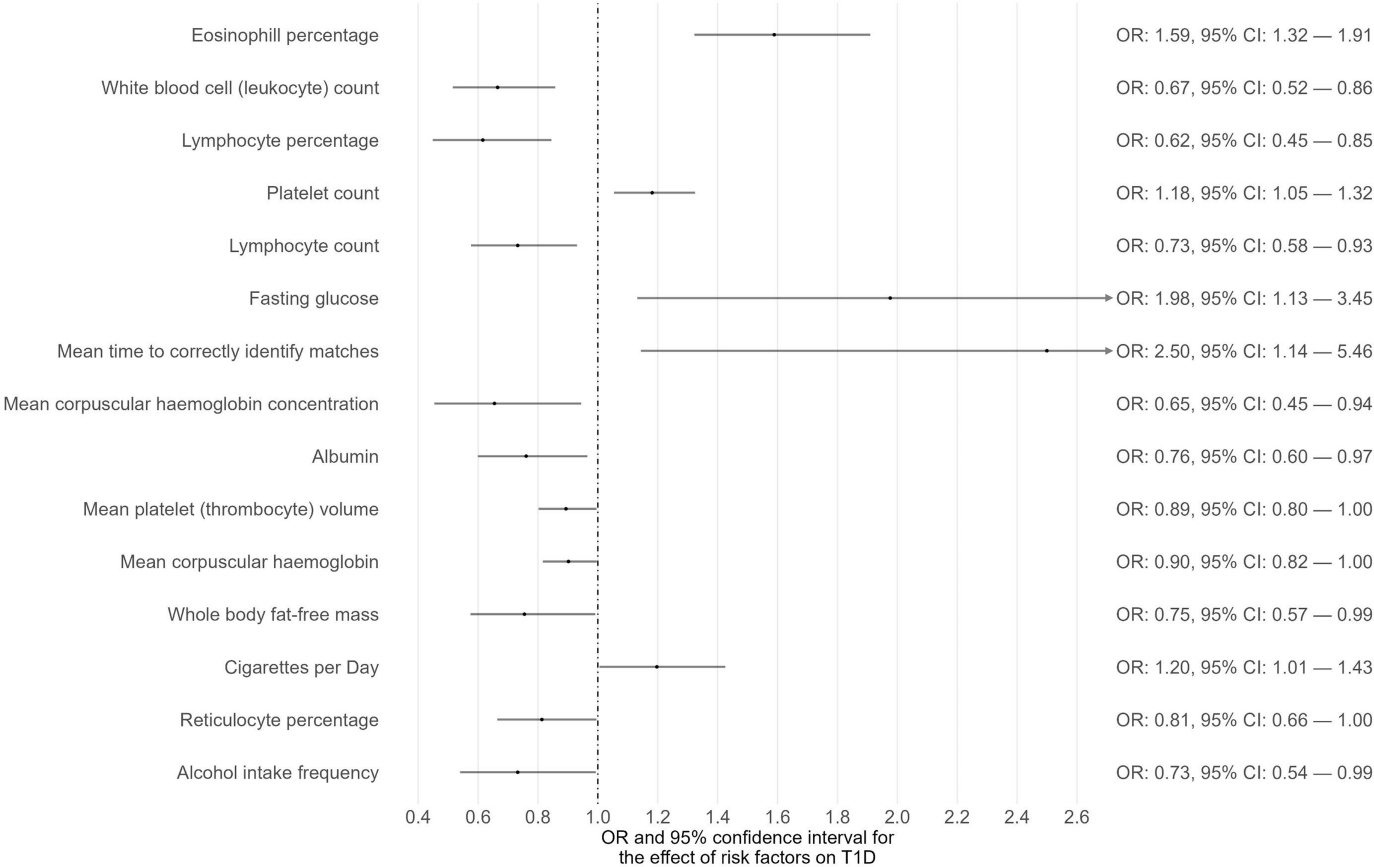
The causal associations between exposures and T1D with suggestive level of significance (*P* < 0.05).

**Figure 2:**
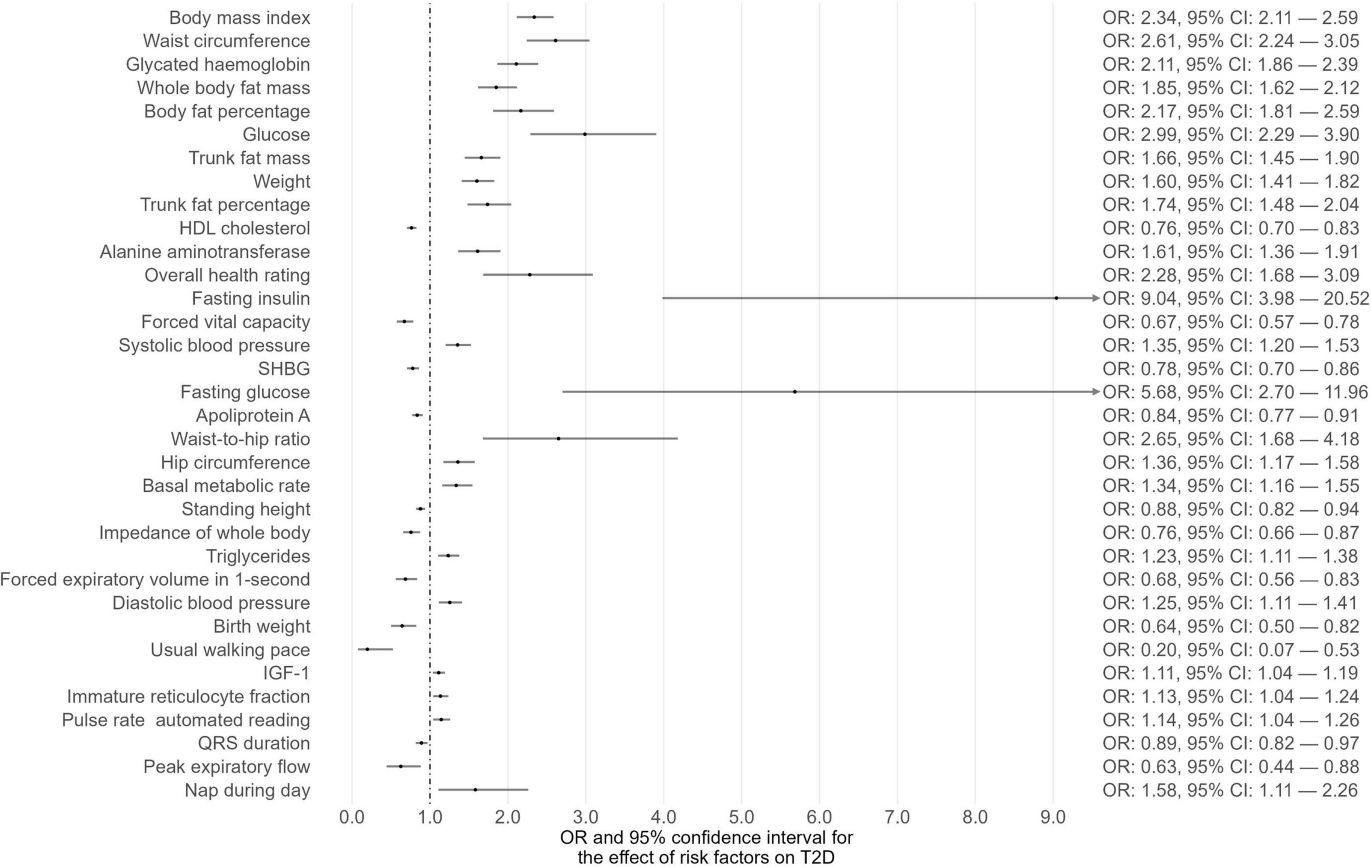
The causal associations between exposures and T2D that survived FDR correction.

**Figure 3:**
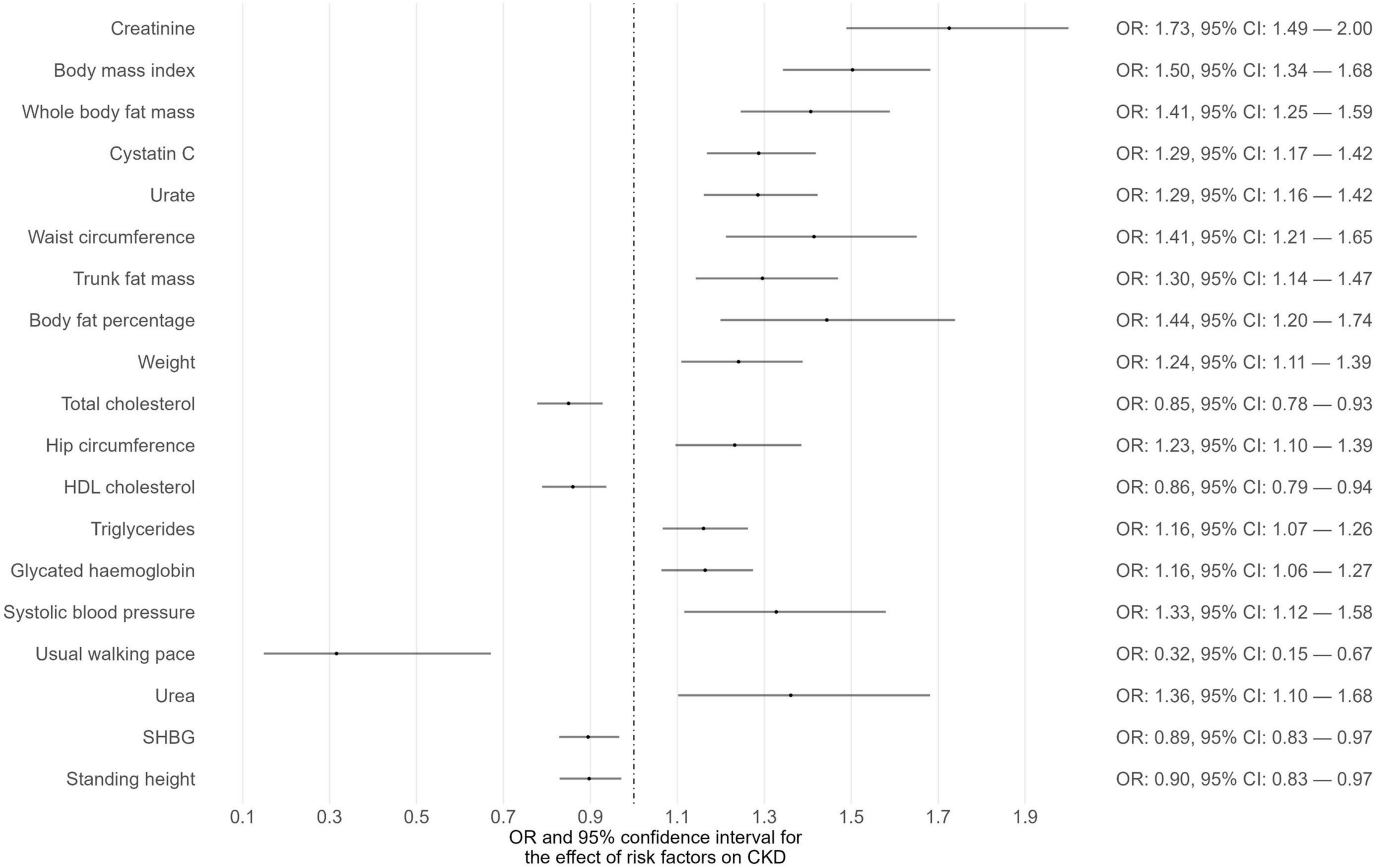
The causal associations between exposures and CKD that survived FDR correction.

Phenome-wide MR analyses were conducted to identify potential risk factors for T1D, T2D, and CKD separately (**Supplementary Tables 1**-**3**). The GWAS summary data of 118 exposures from the IEU OpenGWAS database were incorporated to achieve this. For T1D as the outcome, 15 traits among the 118 exhibited suggestive evidence of association (P < 0.05) (**Figure 1**; **Supplementary Table 1**). Among these 15 traits, only one trait (eosinophil percentage) remained significant after correcting for multiple comparisons using a 5% FDR. Moreover, the associations of eosinophil percentage with T1D were consistent across three different MR methods (IVW, WM, and MR-Egger). For T2D as the outcome, 40 traits among the 118 showed suggestive evidence of association (P < 0.05) (**Supplementary Table 2**). Among these 40 traits, 34 traits (e.g., body mass index, waist circumference, glycated hemoglobin) remained significant after multiple comparison correction with a 5% FDR (**Figure 2**). For CKD as the outcome, 35 traits among the 118 exhibited suggestive evidence of association (P < 0.05) (**Supplementary Table 3**). Among these 35 traits, 19 traits (e.g., creatinine, body mass index, whole-body fat mass) remained significant after correcting for multiple comparisons using a 5% FDR (**Figure 3**).

**Figure 4:**
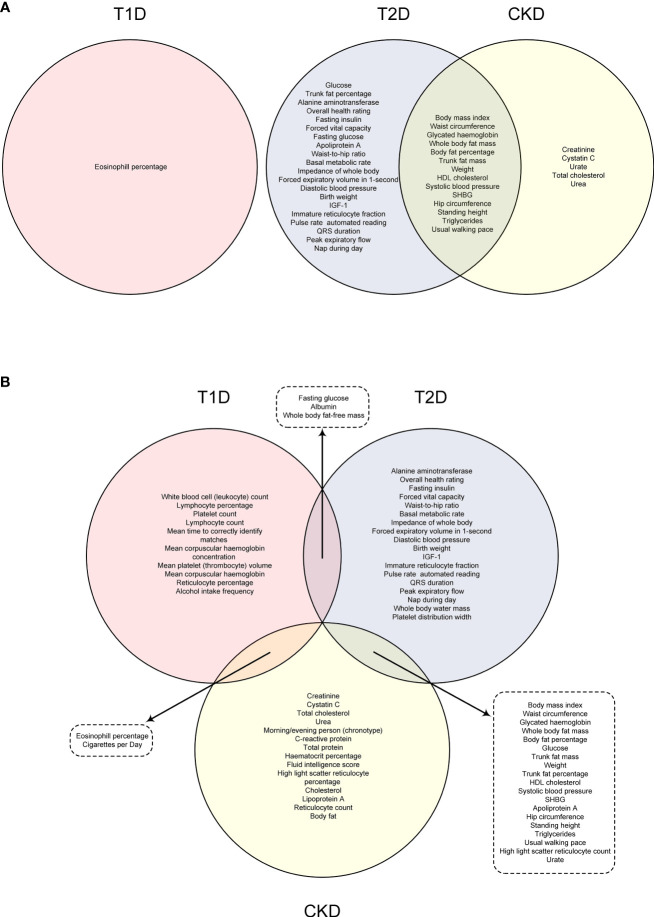
Venn graph indicating the common risk factors among T1D, T2D and CKD under FDR correction **(A)** or with suggestive level of significance (*P* < 0.05) **(B)**.

To demonstrate the shared risk factors between diabetes and CKD, a Venn diagram was created to illustrate the common risk factors of T1D, T2D, and CKD that survived 5% FDR correction (**Figure 4A**) or showed suggestive significance (**Figure 4B**). After correcting for multiple comparisons using a 5% FDR, no common risk factor between T1D and CKD was identified, while 14 common risk factors between T2D and CKD were found, including body mass index, waist circumference, and glycated hemoglobin (**Figure 4A**). If the P-value threshold was relaxed to 0.05, eosinophil percentage and “cigarettes per day” were identified as common risk factors for both T1D and CKD, and 19 exposure traits were found to be common risk factors for both T2D and CKD (**Figure 4B**). However, no risk factor was common to all three disease outcomes (**Figure 4B**).

## Discussion

In the present study, we conducted phenome-wide MR analyses incorporating 118 exposure traits. Our findings revealed that eosinophil percentage exhibited a significant causal association with T1D following correction for multiple comparisons. Additionally, eosinophil percentage showed suggestive evidence of association with CKD, suggesting its potential as a shared risk factor for T1D and CKD. In the case of T2D, 34 traits demonstrated significant associations after correcting for multiple comparisons. Among these 34 traits, 14 were also associated with CKD, indicating the presence of common risk factors between T2D and CKD, predominantly related to obesity, height, blood lipids and sex hormone binding globulin (SHBG), blood pressure, and walking pace.

### High eosinophil levels as a shared risk factor for T1D and CKD

Eosinophils play an important role in the immune response (15). Traditionally, eosinophils have been associated with certain allergic diseases (15). In individuals with T1D, eosinophils are found in pancreatic tissue, and significant differences in eosinophil levels have been observed between T1D patients and healthy individuals (16). Notably, T1D patients exhibit higher percentages of immature eosinophils in circulation compared to healthy individuals (16). Transcriptionally active eosinophils in patients with diabetes indicates their involvement in the complex network of innate immune cells associated with the development of diabetes (17). Eosinophils from individuals with T1D have elevated levels of myeloid alpha-defensins and myeloperoxidase, which play a role in inflammatory and autoimmune diseases (17, 18). Moreover, a study using animal model of diabetes demonstrated a relationship between the expression of defensins in specific tissues and the occurrence of diabetes (17).

Increased levels of eosinophils have also been linked to a higher risk of CKD (19). Eosinophils release a range of inflammatory signaling molecules, which can contribute to chronic inflammatory infiltration in the kidneys (20, 21). Eosinophil presence in the kidneys can promote oxidative stress and contribute to the release of pro-fibrotic factors, causing renal fibrosis (15, 22). These findings support the notion that peripheral eosinophilia and the accumulation of eosinophil-derived cytokines stimulate fibroblast proliferation and contribute to tissue damage, particularly in the kidneys.

### Obesity as a shared risk factor for T2D and CKD

Obesity, characterized by excessive accumulation of body fat that impairs physical and psychosocial health, is a well-known risk factor for various non-communicable diseases (23). T2D is highly associated with obesity, and the prevalence of diabetes which is related to obesity is projected to double by 2025 (24). Adipose tissues in obese individuals increase the release of free fatty acids (FFAs) through enhanced lipolysis, resulting in elevated levels of circulating FFAs. This process promotes muscle and hepatic insulin resistance and impairs insulin secretion by pancreatic β-cells (25, 26).

Obesity has also been identified as a major cause of CKD, independent of body mass index (27). A high BMI is a strong risk factor for the onset and progression of CKD and end-stage renal disease (ESRD) (28, 29). Obesity is associated with several risk factors that contribute to the increased incidence and prevalence of nephrolithiasis, such as abnormal urinary composition and renal hyperfiltration (28, 30). Additionally, obesity-related insulin resistance exacerbates the effects of angiotensin-II, leading to increased proteinuria and the production of inflammatory cytokines, all of which contribute to kidney damage (31). The increased enteral oxalate resulting from insulin resistance in obesity may also predispose individuals to nephrolithiasis (32).

### Central obesity as a shared risk factor for T2D and CKD

Central obesity, featured by an accumulation of fat beyond the normal level in the abdominal region, is a specific type of obesity measured using indices like waist circumference. Individuals with central obesity have a significantly higher likelihood of developing diabetes (33). Excess body fat, especially visceral adipose tissue, is a known risk factor for T2D (34). Central obesity has also been correlated with an increased risk of CKD, regardless of BMI (35). The adipose tissue in the central region secretes various adipokines, including leptin and adiponectin, which influence insulin sensitivity and the regulation of glucose levels. Leptin can promote insulin resistance and downregulate insulin signaling in various cell models (36). Visceral adipose tissue, which is characteristic of central obesity, produces more pro-inflammatory cytokines like interleukin-6 (37). Chronic low-grade inflammation associated with visceral fat accumulation contributes to the development of both T2D and CKD.

### Dyslipidemia as a shared risk factor for T2D and CKD

Dyslipidemia refers to abnormal lipid profiles, and is closely associated with diabetes. Hyperglycemia causes apoptosis of β-cells in the pancreas, and affects the accumulation of oxidized LDLs. Dyslipidemia has a significant impact on the adverse outcomes of diabetes (38). High-density lipoprotein (HDL) exerts several anti-atherogenic effects, including anti-inflammatory, antioxidant, and anti-thrombotic properties (39). More than 75% of patients with T2D have mixed dyslipidemia, featured by low HDL cholesterol levels and high triglycerides levels (40). Low HDL-C levels have been related to impaired β-cell function in individuals with an altered state of fasting glucose levels or glucose tolerance (41). Decreased β-cell survival and secretory function may contribute to the increased risk of T2DM associated with low HDL levels (42).

In the development of CKD, reduced lecithin-cholesterol acyltransferase activity hinders the maturation of lipid-poor precursors of HDL (pre-β HDL) into spherical HDL particles (43). The degraded pre-β HDLs are cleared by the kidneys, leading to decreased apolipoprotein A-I level, which is a component of HDL (44). Thus, altered lipid metabolism and decreased HDL function contribute to the development of CKD.

### Low levels of SHBG as a shared risk factor for T2D and CKD

Reduced levels of SHBG are often seen in insulin-resistant conditions and have been investigated as a predictor of the T2D risk in overweight populations (45). Variants of certain SHBG single-nucleotide polymorphisms (SNPs) are associated with altered SHBG levels and an increased risk of T2D (46). Clinical studies have linked low circulating levels of SHBG to impaired glucose control, suggesting a role for SHBG in maintaining glucose homeostasis (47).

SHBG also potentially reduces sex hormone bioactivity and plays a role in CKD (48). Men with lower SHBG levels have a higher risk of low estimated glomerular filtration rate (eGFR), an indicator of reduced kidney function (49). *In vitro* experiments have shown that SHBG suppresses inflammation, which could be relevant to the association between SHBG and CKD (50). Inflammation and insulin resistance may mediate the link between SHBG and CKD.

### Short stature as a shared risk factor for T2D and CKD

Height plays a role in determining overall health, with associations observed between height and mortality as well as various diseases such as cancers and cardiovascular diseases (51). Numerous studies have indicated a significant link between shorter stature and an elevated risk of developing diabetes. The hazard ratio for developing diabetes gradually increases from the 5th quintile (reference) to the 1st quintile group based on height measurements (52). Furthermore, a comprehensive meta-analysis utilizing random-effects models indicated an inverse relationship between adult height and T2D (51).

There is an negative association of adult height with the incidence of newly diagnosed ESRD as well as all-cause mortality (53). Causal estimates based on eGFR and CKD indicate that taller individuals genetically predisposed to greater height have a lower log-eGFR and a higher risk of developing CKD (54). Short stature may serve as an indicator of insufficient fetal growth during childhood, potentially influencing the development of certain metabolic diseases in adulthood. Further work is necessary to study the biological mechanisms underlying the potential impact of height on the risk of T2D and CKD.

### High blood pressure as a shared risk factor for T2D and CKD

Individuals with T2D have a higher prevalence of elevated blood pressure compared to the general population (55). Hypertension is a known risk factor for individuals with diabetes (56). While high blood pressure is related with an enhanced risk of developing T2D, its independent association with new-onset diabetes is less apparent (57). Resistant hypertension is usually seen in CKD, and increased blood pressure can raise glomerular capillary pressure and the filtration rate (58). Thus, high blood pressure contributes to the development of both T2D and CKD.

### Slow self-reported walking pace as a shared risk factor for T2D and CKD

Walking is the most commonly chosen form of physical activity among older adults (59). Numerous studies have shown that indicators of physical capability, such as walking pace, are correlated with a range of health consequences (60). Gait speed has been identified as a strong indicator of the extent of functional changes in the olders (61). Combining walking pace with grip strength could be a practical method for identifying individuals at a higher risk of developing T2D (62).

Numerous studies have also provided evidence that the gait speed test is a reliable measure for assessing the risk of all-cause mortality in individuals with CKD (63). Additionally, impaired ability to engage in physical exercise has been revealed to be an indicator of survival among ambulatory patients with ESRD (64). Participants who exhibited lower walking speed or were unable to walk demonstrated higher level of muscle mass deterioration, leading to a decreased function and heightened vulnerability to harmful conditions related to CKD (63).

### Strengths and limitations

The current study benefits from the use of a MR design, which helps reduce biases stemming from residual confounding and reverse causality. To account for potential issues like horizontal pleiotropy and instrument strength, various methods of MR, including the WM method and MR-Egger, were utilized for sensitivity analyses. However, there are several limitations to consider in this study. Firstly, the inclusion of a relatively large number of traits increased the challenge of correcting for multiple comparisons. Secondly, horizontal pleiotropy, which is commonly observed in MR analyses, may have introduced bias into the findings of this study.

## Conclusion

This research has uncovered the eosinophil percentage as a potential common risk factor for both T1D and CKD, while also identifying several traits, such as obesity and blood lipids, as shared risk factors for T2D and CKD. Focusing on precise risk reduction initiatives holds the potential to simultaneously impact both T2D and CKD. This strategy has the capability to bring about substantial advantages for the overall well-being of the public and enhance the quality of life for individuals who are affected by these diseases. Understanding the shared risk factors and their interplay is also crucial for the effective development of public health interventions.

## Data availability statement

The original contributions presented in the study are included in the article/**Supplementary Material**. Further inquiries can be directed to the corresponding author.

## Author contributions

SZ: Writing – original draft, Formal Analysis, Methodology, Software. YL: Writing – original draft, Conceptualization, Data curation. CS: Writing – original draft, Investigation, Project administration, Supervision, Writing – review & editing.
