# Supplementary material for: Assessment of common risk factors of diabetes and chronic kidney disease: a Mendelian randomization study
Source: Front Endocrinol (Lausanne). 2023 Sep 13;14:1265719. doi: 10.3389/fendo.2023.1265719 (PMC10535100; doi:10.3389/fendo.2023.1265719)
Supplement: Supplementary file 1 [file Table_1.pdf]

Supplementary Table 1. MR analyses of the effect of exposures on T1D

Trait	Trait ID	Method	nsnp	Beta	SE	P
Adiponectin	ieu-a-1	IVW	14	0.1554	0.1024	1.29E-01
Adiponectin	ieu-a-1	MR Egger	14	0.0086	0.1341	9.50E-01
Adiponectin	ieu-a-1	WM	14	0.0843	0.1126	4.54E-01
Alanine aminotransferase	ukb-d-30620_irnt	IVW	166	0.2301	0.1726	1.82E-01
Alanine aminotransferase	ukb-d-30620_irnt	MR Egger	166	0.2038	0.3204	5.26E-01
Alanine aminotransferase	ukb-d-30620_irnt	WM	166	0.1282	0.1363	3.47E-01
Albumin	ukb-d-30600_irnt	IVW	171	-0.2736	0.1215	2.43E-02
Albumin	ukb-d-30600_irnt	MR Egger	171	-0.3395	0.2399	1.59E-01
Albumin	ukb-d-30600_irnt	WM	171	-0.3361	0.1390	1.56E-02
Alcohol intake frequency	ukb-a-25	IVW	40	-0.3115	0.1555	4.51E-02
Alcohol intake frequency	ukb-a-25	MR Egger	40	0.0936	0.5979	8.76E-01
Alcohol intake frequency	ukb-a-25	WM	40	-0.2162	0.2267	3.40E-01
Alcohol intake versus 10 years previously	ukb-a-32	IVW	7	-1.2018	0.8199	1.43E-01
Alcohol intake versus 10 years previously	ukb-a-32	MR Egger	7	-2.1548	10.3584	8.43E-01
Alcohol intake versus 10 years previously	ukb-a-32	WM	7	-0.8423	1.0915	4.40E-01
Alcoholic drinks per week	ieu-b-73	IVW	32	0.6958	0.5267	1.86E-01
Alcoholic drinks per week	ieu-b-73	MR Egger	32	-0.1845	1.6279	9.11E-01
Alcoholic drinks per week	ieu-b-73	WM	32	0.3174	0.4999	5.25E-01
Alkaline phosphatase	ukb-d-30610_irnt	IVW	261	0.0103	0.1197	9.31E-01
Alkaline phosphatase	ukb-d-30610_irnt	MR Egger	261	-0.2393	0.1980	2.28E-01
Alkaline phosphatase	ukb-d-30610_irnt	WM	261	0.0038	0.0969	9.69E-01
Apoliprotein A	ukb-d-30630_irnt	IVW	198	-0.1318	0.1219	2.79E-01
Apoliprotein A	ukb-d-30630_irnt	MR Egger	198	0.0120	0.1866	9.49E-01
Apoliprotein A	ukb-d-30630_irnt	WM	198	-0.0811	0.1060	4.44E-01
Apoliprotein B	ukb-d-30640_irnt	IVW	132	-0.0589	0.0793	4.57E-01
Apoliprotein B	ukb-d-30640_irnt	MR Egger	132	-0.1132	0.1146	3.25E-01
Apoliprotein B	ukb-d-30640_irnt	WM	132	-0.1848	0.0983	6.00E-02
Aspartate aminotransferase	ukb-d-30650_irnt	IVW	197	-0.0895	0.1242	4.71E-01
Aspartate aminotransferase	ukb-d-30650_irnt	MR Egger	197	0.0174	0.2322	9.40E-01
Aspartate aminotransferase	ukb-d-30650_irnt	WM	197	0.1019	0.1318	4.40E-01
Basal metabolic rate	ukb-a-268	IVW	355	-0.0780	0.1486	6.00E-01
Basal metabolic rate	ukb-a-268	MR Egger	355	0.1968	0.3688	5.94E-01
Basal metabolic rate	ukb-a-268	WM	355	-0.0068	0.1417	9.62E-01
Basophill percentage	ukb-d-30220_irnt	IVW	93	0.2920	0.2151	1.75E-01
Basophill percentage	ukb-d-30220_irnt	MR Egger	93	0.0417	0.4054	9.18E-01
Basophill percentage	ukb-d-30220_irnt	WM	93	0.0594	0.1509	6.94E-01
Birth weight	ukb-a-198	IVW	75	-0.1481	0.1449	3.07E-01
Birth weight	ukb-a-198	MR Egger	75	-0.1087	0.5069	8.31E-01
Birth weight	ukb-a-198	WM	75	0.1269	0.1764	4.72E-01
Birth weight of first child	ukb-a-318	IVW	41	0.2104	0.2300	3.60E-01
Birth weight of first child	ukb-a-318	MR Egger	41	-0.3022	0.8498	7.24E-01
Birth weight of first child	ukb-a-318	WM	41	0.3170	0.1859	8.82E-02

Body fat	ieu-a-999	IVW	10	-0.1622	0.3810	6.70E-01
Body fat	ieu-a-999	MR Egger	10	0.1312	1.8385	9.45E-01
Body fat	ieu-a-999	WM	10	-0.2149	0.3509	5.40E-01
Body fat percentage	ukb-a-264	IVW	238	0.1473	0.1626	3.65E-01
Body fat percentage	ukb-a-264	MR Egger	238	0.3291	0.5985	5.83E-01
Body fat percentage	ukb-a-264	WM	238	0.0356	0.1716	8.36E-01
body mass index	ieu-b-40	IVW	476	-0.0008	0.0924	9.93E-01
body mass index	ieu-b-40	MR Egger	476	0.5138	0.2434	3.53E-02
body mass index	ieu-b-40	WM	476	0.0849	0.1209	4.83E-01
Calcium	ukb-d-30680_irnt	IVW	172	-0.1513	0.1304	2.46E-01
Calcium	ukb-d-30680_irnt	MR Egger	172	-0.1066	0.2413	6.59E-01
Calcium	ukb-d-30680_irnt	WM	172	0.0141	0.1446	9.22E-01
Cholesterol	ukb-d-30690_irnt	IVW	139	-0.2318	0.1809	2.00E-01
Cholesterol	ukb-d-30690_irnt	MR Egger	139	-0.0285	0.2949	9.23E-01
Cholesterol	ukb-d-30690_irnt	WM	139	-0.0231	0.1101	8.34E-01
Cigarettes per Day	ieu-b-25	IVW	22	0.1799	0.0891	4.35E-02
Cigarettes per Day	ieu-b-25	MR Egger	22	0.2373	0.1559	1.44E-01
Cigarettes per Day	ieu-b-25	WM	22	0.2358	0.1182	4.61E-02
C-reactive protein	ukb-d-30710_irnt	IVW	170	0.1002	0.2181	6.46E-01
C-reactive protein	ukb-d-30710_irnt	MR Egger	170	0.1114	0.3018	7.12E-01
C-reactive protein	ukb-d-30710_irnt	WM	170	-0.0965	0.0804	2.30E-01
Creatinine	ukb-d-30700_irnt	IVW	268	0.0500	0.0959	6.02E-01
Creatinine	ukb-d-30700_irnt	MR Egger	268	0.0395	0.2224	8.59E-01
Creatinine	ukb-d-30700_irnt	WM	268	0.0284	0.1273	8.24E-01
Creatinine (enzymatic) in urine	ukb-a-333	IVW	21	-0.2730	0.4715	5.63E-01
Creatinine (enzymatic) in urine	ukb-a-333	MR Egger	21	0.9236	1.9921	6.48E-01
Creatinine (enzymatic) in urine	ukb-a-333	WM	21	-0.0736	0.4965	8.82E-01
Current tobacco smoking	ukb-a-16	IVW	16	-0.6034	0.8480	4.77E-01
Current tobacco smoking	ukb-a-16	MR Egger	16	3.6102	4.0040	3.82E-01
Current tobacco smoking	ukb-a-16	WM	16	0.6378	0.9854	5.17E-01
Cystatin C	ukb-d-30720_irnt	IVW	261	0.0533	0.0719	4.58E-01
Cystatin C	ukb-d-30720_irnt	MR Egger	261	-0.0860	0.0986	3.83E-01
Cystatin C	ukb-d-30720_irnt	WM	261	-0.0770	0.0733	2.94E-01
Daytime dozing / sleeping (narcolepsy)	ukb-a-15	IVW	19	-0.8681	0.8771	3.22E-01
Daytime dozing / sleeping (narcolepsy)	ukb-a-15	MR Egger	19	3.3518	4.2589	4.42E-01
Daytime dozing / sleeping (narcolepsy)	ukb-a-15	WM	19	-0.1278	1.0461	9.03E-01
Diastolic blood pressure	ukb-a-359	IVW	165	0.0037	0.1391	9.79E-01
Diastolic blood pressure	ukb-a-359	MR Egger	165	0.2578	0.4880	5.98E-01
Diastolic blood pressure	ukb-a-359	WM	165	0.1434	0.1570	3.61E-01
Direct bilirubin	ukb-d-30660_irnt	IVW	63	0.0167	0.0424	6.94E-01
Direct bilirubin	ukb-d-30660_irnt	MR Egger	63	0.0232	0.0469	6.23E-01
Direct bilirubin	ukb-d-30660_irnt	WM	63	0.0474	0.0379	2.11E-01
Drive faster than motorway speed limit	ukb-a-8	IVW	13	-1.3713	2.1120	5.16E-01
Drive faster than motorway speed limit	ukb-a-8	MR Egger	13	13.6063	10.8054	2.34E-01
Drive faster than motorway speed limit	ukb-a-8	WM	13	1.0904	0.7864	1.66E-01

Eosinophill percentage	ukb-d-30210_irnt	IVW	266	0.4632	0.0938	7.90E-07
Eosinophill percentage	ukb-d-30210_irnt	MR Egger	266	0.4199	0.1928	3.03E-02
Eosinophill percentage	ukb-d-30210_irnt	WM	266	0.3301	0.1026	1.29E-03
Fasting glucose	ieu-b-114	IVW	30	0.6813	0.2845	1.67E-02
Fasting glucose	ieu-b-114	MR Egger	30	-0.2051	0.5898	7.31E-01
Fasting glucose	ieu-b-114	WM	30	0.4199	0.2915	1.50E-01
Fasting insulin	ieu-b-116	IVW	14	0.1419	0.4544	7.55E-01
Fasting insulin	ieu-b-116	MR Egger	14	-0.6825	2.4384	7.84E-01
Fasting insulin	ieu-b-116	WM	14	0.1061	0.5471	8.46E-01
Fluid intelligence score	ukb-a-196	IVW	42	0.0748	0.1359	5.82E-01
Fluid intelligence score	ukb-a-196	MR Egger	42	0.0956	0.6366	8.81E-01
Fluid intelligence score	ukb-a-196	WM	42	-0.1086	0.1014	2.84E-01
Forced expiratory volume in 1-second (FEV1)	ukb-a-337	IVW	144	-0.4230	0.2423	8.09E-02
Forced expiratory volume in 1-second (FEV1)	ukb-a-337	MR Egger	144	-0.4815	0.8084	5.52E-01
Forced expiratory volume in 1-second (FEV1)	ukb-a-337	WM	144	0.0677	0.1950	7.29E-01
Forced vital capacity (FVC)	ukb-a-336	IVW	202	-0.2499	0.2286	2.74E-01
Forced vital capacity (FVC)	ukb-a-336	MR Egger	202	-1.7842	0.6574	7.23E-03
Forced vital capacity (FVC)	ukb-a-336	WM	202	0.0774	0.1741	6.57E-01
Gamma glutamyltransferase	ukb-d-30730_irnt	IVW	227	0.0592	0.0760	4.36E-01
Gamma glutamyltransferase	ukb-d-30730_irnt	MR Egger	227	0.0963	0.1220	4.31E-01
Gamma glutamyltransferase	ukb-d-30730_irnt	WM	227	0.0863	0.1078	4.23E-01
Getting up in morning	ukb-a-10	IVW	36	0.0521	0.3319	8.75E-01
Getting up in morning	ukb-a-10	MR Egger	36	1.4668	1.1970	2.29E-01
Getting up in morning	ukb-a-10	WM	36	0.5028	0.4896	3.04E-01
Glucose	ukb-d-30740_irnt	IVW	86	0.2440	0.3182	4.43E-01
Glucose	ukb-d-30740_irnt	MR Egger	86	0.0974	0.5200	8.52E-01
Glucose	ukb-d-30740_irnt	WM	86	0.1748	0.1516	2.49E-01
Glycated haemoglobin	ukb-d-30750_irnt	IVW	249	0.1512	0.1629	3.53E-01
Glycated haemoglobin	ukb-d-30750_irnt	MR Egger	249	0.2937	0.2755	2.87E-01
Glycated haemoglobin	ukb-d-30750_irnt	WM	249	0.1823	0.1135	1.08E-01
Haematocrit percentage	ukb-d-30030_irnt	IVW	211	-0.1236	0.1096	2.59E-01
Haematocrit percentage	ukb-d-30030_irnt	MR Egger	211	-0.0786	0.2340	7.37E-01
Haematocrit percentage	ukb-d-30030_irnt	WM	211	-0.0040	0.1570	9.80E-01
Haemoglobin concentration	ukb-d-30020_irnt	IVW	232	-0.1614	0.1100	1.42E-01
Haemoglobin concentration	ukb-d-30020_irnt	MR Egger	232	-0.1509	0.2277	5.08E-01
Haemoglobin concentration	ukb-d-30020_irnt	WM	232	-0.1739	0.1467	2.36E-01
HDL cholesterol	ukb-d-30760_irnt	IVW	222	0.0474	0.1133	6.76E-01
HDL cholesterol	ukb-d-30760_irnt	MR Egger	222	0.0045	0.1674	9.79E-01
HDL cholesterol	ukb-d-30760_irnt	WM	222	-0.0603	0.1002	5.47E-01
Heart rate	ieu-a-1056	IVW	14	-0.0224	0.0163	1.70E-01
Heart rate	ieu-a-1056	MR Egger	14	-0.0610	0.0644	3.62E-01
Heart rate	ieu-a-1056	WM	14	-0.0139	0.0211	5.10E-01
Heel bone mineral density (BMD) T-score	ukb-a-500	IVW	232	0.0271	0.0555	6.25E-01
Heel bone mineral density (BMD) T-score	ukb-a-500	MR Egger	232	0.2090	0.1064	5.07E-02
Heel bone mineral density (BMD) T-score	ukb-a-500	WM	232	0.1719	0.0809	3.37E-02

High light scatter reticulocyte count	ukb-d-30300_irnt	IVW	250	-0.0325	0.1365	8.12E-01
High light scatter reticulocyte count	ukb-d-30300_irnt	MR Egger	250	-0.3099	0.2655	2.44E-01
High light scatter reticulocyte count	ukb-d-30300_irnt	WM	250	-0.0327	0.1080	7.62E-01
High light scatter reticulocyte percentage	ukb-d-30290_irnt	IVW	260	-0.1266	0.0891	1.55E-01
High light scatter reticulocyte percentage	ukb-d-30290_irnt	MR Egger	260	-0.1272	0.1705	4.56E-01
High light scatter reticulocyte percentage	ukb-d-30290_irnt	WM	260	-0.0181	0.1024	8.60E-01
Hip circumference	ukb-a-388	IVW	270	0.0225	0.1068	8.33E-01
Hip circumference	ukb-a-388	MR Egger	270	0.6534	0.3018	3.13E-02
Hip circumference	ukb-a-388	WM	270	0.0603	0.1238	6.26E-01
IGF-1	ukb-d-30770_irnt	IVW	288	0.0396	0.0778	6.10E-01
IGF-1	ukb-d-30770_irnt	MR Egger	288	-0.0361	0.1521	8.12E-01
IGF-1	ukb-d-30770_irnt	WM	288	-0.0315	0.1164	7.87E-01
Immature reticulocyte fraction	ukb-d-30280_irnt	IVW	181	-0.0723	0.1153	5.30E-01
Immature reticulocyte fraction	ukb-d-30280_irnt	MR Egger	181	0.1826	0.2098	3.85E-01
Immature reticulocyte fraction	ukb-d-30280_irnt	WM	181	0.1174	0.1166	3.14E-01
Impedance of whole body	ukb-a-269	IVW	333	0.1785	0.1171	1.27E-01
Impedance of whole body	ukb-a-269	MR Egger	333	0.1971	0.3290	5.50E-01
Impedance of whole body	ukb-a-269	WM	333	-0.0190	0.1452	8.96E-01
Job involves mainly walking or standing	ukb-a-502	IVW	7	-0.3597	0.4642	4.38E-01
Job involves mainly walking or standing	ukb-a-502	MR Egger	7	1.4061	5.0256	7.91E-01
Job involves mainly walking or standing	ukb-a-502	WM	7	-0.4302	0.5241	4.12E-01
LDL direct	ukb-d-30780_irnt	IVW	128	-0.0706	0.0999	4.80E-01
LDL direct	ukb-d-30780_irnt	MR Egger	128	-0.0608	0.1502	6.86E-01
LDL direct	ukb-d-30780_irnt	WM	128	-0.0476	0.1165	6.83E-01
Length of menstrual cycle	ukb-a-351	IVW	6	-0.1320	0.1687	4.34E-01
Length of menstrual cycle	ukb-a-351	MR Egger	6	-0.4740	0.3761	2.76E-01
Length of menstrual cycle	ukb-a-351	WM	6	-0.2396	0.1998	2.31E-01
Lipoprotein A	ukb-d-30790_irnt	IVW	17	-0.0147	0.0310	6.34E-01
Lipoprotein A	ukb-d-30790_irnt	MR Egger	17	-0.0418	0.0379	2.87E-01
Lipoprotein A	ukb-d-30790_irnt	WM	17	-0.0322	0.0356	3.66E-01
Lymphocyte count	ukb-d-30120_irnt	IVW	279	-0.3116	0.1223	1.08E-02
Lymphocyte count	ukb-d-30120_irnt	MR Egger	279	-0.4785	0.2913	1.02E-01
Lymphocyte count	ukb-d-30120_irnt	WM	279	-0.1445	0.1129	2.00E-01
Lymphocyte percentage	ukb-d-30180_irnt	IVW	238	-0.4849	0.1615	2.68E-03
Lymphocyte percentage	ukb-d-30180_irnt	MR Egger	238	-0.8255	0.3613	2.32E-02
Lymphocyte percentage	ukb-d-30180_irnt	WM	238	-0.2488	0.1314	5.82E-02
Mean corpuscular haemoglobin	ukb-d-30050_irnt	IVW	295	-0.1033	0.0510	4.30E-02
Mean corpuscular haemoglobin	ukb-d-30050_irnt	MR Egger	295	-0.1273	0.0828	1.25E-01
Mean corpuscular haemoglobin	ukb-d-30050_irnt	WM	295	-0.0457	0.0795	5.65E-01
Mean corpuscular haemoglobin concentration	ukb-d-30060_irnt	IVW	84	-0.4237	0.1868	2.33E-02
Mean corpuscular haemoglobin concentration	ukb-d-30060_irnt	MR Egger	84	-0.6577	0.3710	8.00E-02
Mean corpuscular haemoglobin concentration	ukb-d-30060_irnt	WM	84	-0.1119	0.1624	4.91E-01
Mean corpuscular volume	ukb-d-30040_irnt	IVW	301	-0.1039	0.0592	7.94E-02
Mean corpuscular volume	ukb-d-30040_irnt	MR Egger	301	-0.2122	0.1009	3.63E-02
Mean corpuscular volume	ukb-d-30040_irnt	WM	301	-0.0442	0.0852	6.04E-01

Mean platelet (thrombocyte) volume	ukb-d-30100_irnt	IVW	356	-0.1126	0.0552	4.13E-02
Mean platelet (thrombocyte) volume	ukb-d-30100_irnt	MR Egger	356	-0.0983	0.0828	2.36E-01
Mean platelet (thrombocyte) volume	ukb-d-30100_irnt	WM	356	0.0858	0.0648	1.86E-01
Mean reticulocyte volume	ukb-d-30260_irnt	IVW	268	0.1194	0.0910	1.89E-01
Mean reticulocyte volume	ukb-d-30260_irnt	MR Egger	268	0.1118	0.1646	4.98E-01
Mean reticulocyte volume	ukb-d-30260_irnt	WM	268	-0.0143	0.0833	8.64E-01
Mean sphered cell volume	ukb-d-30270_irnt	IVW	276	-0.1459	0.0979	1.36E-01
Mean sphered cell volume	ukb-d-30270_irnt	MR Egger	276	-0.0283	0.1727	8.70E-01
Mean sphered cell volume	ukb-d-30270_irnt	WM	276	-0.0115	0.0826	8.89E-01
Mean time to correctly identify matches	ukb-a-199	IVW	26	0.9161	0.3989	2.17E-02
Mean time to correctly identify matches	ukb-a-199	MR Egger	26	-1.6198	2.6126	5.41E-01
Mean time to correctly identify matches	ukb-a-199	WM	26	0.4132	0.4383	3.46E-01
Microalbumin in urine	ukb-d-30500_irnt	IVW	4	-0.2390	0.3862	5.36E-01
Microalbumin in urine	ukb-d-30500_irnt	MR Egger	4	-0.9204	0.7533	3.46E-01
Microalbumin in urine	ukb-d-30500_irnt	WM	4	-0.5003	0.4568	2.73E-01
Monocyte count	ukb-d-30130_irnt	IVW	277	-0.0913	0.1533	5.52E-01
Monocyte count	ukb-d-30130_irnt	MR Egger	277	-0.4325	0.2476	8.18E-02
Monocyte count	ukb-d-30130_irnt	WM	277	0.0576	0.0999	5.64E-01
Monocyte percentage	ukb-d-30190_irnt	IVW	257	0.1007	0.0751	1.80E-01
Monocyte percentage	ukb-d-30190_irnt	MR Egger	257	-0.0949	0.1243	4.46E-01
Monocyte percentage	ukb-d-30190_irnt	WM	257	0.1505	0.0921	1.02E-01
Morning/evening person (chronotype)	ukb-a-11	IVW	80	0.0993	0.1894	6.00E-01
Morning/evening person (chronotype)	ukb-a-11	MR Egger	80	-0.4091	0.4389	3.54E-01
Morning/evening person (chronotype)	ukb-a-11	WM	80	-0.2181	0.2638	4.08E-01
Nap during day	ukb-a-12	IVW	47	0.3708	0.3481	2.87E-01
Nap during day	ukb-a-12	MR Egger	47	1.0318	1.3147	4.37E-01
Nap during day	ukb-a-12	WM	47	0.4261	0.5083	4.02E-01
Neuroticism	ieu-a-1007	IVW	9	0.2662	0.4494	5.54E-01
Neuroticism	ieu-a-1007	MR Egger	9	0.7153	3.8246	8.57E-01
Neuroticism	ieu-a-1007	WM	9	0.3297	0.5311	5.35E-01
Neuroticism score	ukb-a-230	IVW	62	0.0699	0.0890	4.32E-01
Neuroticism score	ukb-a-230	MR Egger	62	0.0252	0.5275	9.62E-01
Neuroticism score	ukb-a-230	WM	62	-0.0489	0.0817	5.49E-01
Neutrophill count	ukb-d-30140_irnt	IVW	239	-0.1206	0.1226	3.25E-01
Neutrophill count	ukb-d-30140_irnt	MR Egger	239	-0.1472	0.2607	5.73E-01
Neutrophill count	ukb-d-30140_irnt	WM	239	-0.0956	0.1293	4.60E-01
Neutrophill percentage	ukb-d-30200_irnt	IVW	238	0.1040	0.1339	4.37E-01
Neutrophill percentage	ukb-d-30200_irnt	MR Egger	238	0.1367	0.3034	6.53E-01
Neutrophill percentage	ukb-d-30200_irnt	WM	238	-0.0312	0.1347	8.17E-01
Overall health rating	ukb-a-251	IVW	49	-0.1653	0.3949	6.76E-01
Overall health rating	ukb-a-251	MR Egger	49	-0.2231	2.3600	9.25E-01
Overall health rating	ukb-a-251	WM	49	-0.1137	0.4776	8.12E-01
Past tobacco smoking	ukb-a-17	IVW	40	0.0360	0.3029	9.06E-01
Past tobacco smoking	ukb-a-17	MR Egger	40	-0.0413	1.2301	9.73E-01
Past tobacco smoking	ukb-a-17	WM	40	-0.2413	0.2772	3.84E-01

Peak expiratory flow (PEF)	ukb-a-338	IVW	78	-0.4597	0.3562	1.97E-01
Peak expiratory flow (PEF)	ukb-a-338	MR Egger	78	-2.3490	1.2759	6.95E-02
Peak expiratory flow (PEF)	ukb-a-338	WM	78	0.1044	0.2853	7.14E-01
Phosphate	ukb-d-30810_irnt	IVW	134	-0.0659	0.1044	5.28E-01
Phosphate	ukb-d-30810_irnt	MR Egger	134	-0.1575	0.1724	3.63E-01
Phosphate	ukb-d-30810_irnt	WM	134	-0.2268	0.1316	8.49E-02
Platelet count	ukb-d-30080_irnt	IVW	350	0.1668	0.0583	4.26E-03
Platelet count	ukb-d-30080_irnt	MR Egger	350	0.3701	0.1035	4.00E-04
Platelet count	ukb-d-30080_irnt	WM	350	-0.0296	0.0827	7.20E-01
Platelet crit	ukb-d-30090_irnt	IVW	317	0.1169	0.0811	1.49E-01
Platelet crit	ukb-d-30090_irnt	MR Egger	317	0.2690	0.1449	6.44E-02
Platelet crit	ukb-d-30090_irnt	WM	317	0.0890	0.0991	3.69E-01
Platelet distribution width	ukb-d-30110_irnt	IVW	283	-0.0297	0.0499	5.52E-01
Platelet distribution width	ukb-d-30110_irnt	MR Egger	283	-0.0182	0.0736	8.05E-01
Platelet distribution width	ukb-d-30110_irnt	WM	283	-0.0252	0.0799	7.52E-01
Potassium in urine	ukb-a-334	IVW	9	-1.9608	1.8977	3.02E-01
Potassium in urine	ukb-a-334	MR Egger	9	-26.4790	10.1891	3.55E-02
Potassium in urine	ukb-a-334	WM	9	-0.2108	0.7496	7.79E-01
Pulse rate	ukb-a-3	IVW	189	-0.1770	0.0985	7.23E-02
Pulse rate	ukb-a-3	MR Egger	189	-0.0327	0.2327	8.88E-01
Pulse rate	ukb-a-3	WM	189	-0.0524	0.1280	6.82E-01
QRS duration	ukb-d-12340_irnt	IVW	5	-0.0104	0.1882	9.56E-01
QRS duration	ukb-d-12340_irnt	MR Egger	5	0.5338	0.9083	5.98E-01
QRS duration	ukb-d-12340_irnt	WM	5	0.0414	0.1674	8.05E-01
Red blood cell (erythrocyte) count	ukb-d-30010_irnt	IVW	293	-0.0697	0.0968	4.72E-01
Red blood cell (erythrocyte) count	ukb-d-30010_irnt	MR Egger	293	-0.0434	0.1813	8.11E-01
Red blood cell (erythrocyte) count	ukb-d-30010_irnt	WM	293	0.0344	0.1347	7.98E-01
Red blood cell (erythrocyte) distribution width	ukb-d-30070_irnt	IVW	246	0.0857	0.0651	1.88E-01
Red blood cell (erythrocyte) distribution width	ukb-d-30070_irnt	MR Egger	246	0.2309	0.1159	4.76E-02
Red blood cell (erythrocyte) distribution width	ukb-d-30070_irnt	WM	246	0.1021	0.0857	2.33E-01
Reticulocyte count	ukb-d-30250_irnt	IVW	237	-0.0479	0.0784	5.41E-01
Reticulocyte count	ukb-d-30250_irnt	MR Egger	237	-0.1130	0.1448	4.36E-01
Reticulocyte count	ukb-d-30250_irnt	WM	237	-0.1743	0.0987	7.74E-02
Reticulocyte percentage	ukb-d-30240_irnt	IVW	227	-0.2070	0.1032	4.50E-02
Reticulocyte percentage	ukb-d-30240_irnt	MR Egger	227	-0.3170	0.1933	1.02E-01
Reticulocyte percentage	ukb-d-30240_irnt	WM	227	-0.1549	0.1059	1.43E-01
Serum cystatin C (eGFRcys)	ieu-a-1106	IVW	5	0.2459	1.0743	8.19E-01
Serum cystatin C (eGFRcys)	ieu-a-1106	MR Egger	5	1.0546	1.7383	5.87E-01
Serum cystatin C (eGFRcys)	ieu-a-1106	WM	5	0.4681	0.3878	2.27E-01
SHBG	ukb-d-30830_irnt	IVW	221	-0.0237	0.0945	8.02E-01
SHBG	ukb-d-30830_irnt	MR Egger	221	-0.0039	0.1443	9.78E-01
SHBG	ukb-d-30830_irnt	WM	221	0.0177	0.0976	8.56E-01
Sitting height	ukb-a-195	IVW	411	-0.0331	0.0943	7.25E-01
Sitting height	ukb-a-195	MR Egger	411	-0.0352	0.2347	8.81E-01
Sitting height	ukb-a-195	WM	411	0.0722	0.1059	4.96E-01

Sleep duration	ukb-a-9	IVW	41	-0.2100	0.3697	5.70E-01
Sleep duration	ukb-a-9	MR Egger	41	1.3256	1.4907	3.79E-01
Sleep duration	ukb-a-9	WM	41	0.3217	0.4862	5.08E-01
Sleeplessness / insomnia	ukb-a-13	IVW	28	-0.1521	0.4150	7.14E-01
Sleeplessness / insomnia	ukb-a-13	MR Egger	28	0.2270	1.2042	8.52E-01
Sleeplessness / insomnia	ukb-a-13	WM	28	0.2874	0.5975	6.31E-01
Sodium in urine	ukb-a-335	IVW	29	-0.4767	0.3602	1.86E-01
Sodium in urine	ukb-a-335	MR Egger	29	-0.0488	1.6552	9.77E-01
Sodium in urine	ukb-a-335	WM	29	-0.3240	0.4072	4.26E-01
Standing height	ukb-a-389	IVW	564	-0.0206	0.0631	7.44E-01
Standing height	ukb-a-389	MR Egger	564	0.0692	0.1378	6.16E-01
Standing height	ukb-a-389	WM	564	0.0516	0.0882	5.58E-01
Systolic blood pressure	ukb-a-360	IVW	145	0.1064	0.1528	4.86E-01
Systolic blood pressure	ukb-a-360	MR Egger	145	-0.1301	0.5107	7.99E-01
Systolic blood pressure	ukb-a-360	WM	145	0.0609	0.1723	7.24E-01
telomere length	ieu-b-4879	IVW	129	-0.0530	0.1262	6.74E-01
telomere length	ieu-b-4879	MR Egger	129	0.1431	0.2220	5.20E-01
telomere length	ieu-b-4879	WM	129	-0.0430	0.1529	7.79E-01
Testosterone	ukb-d-30850_irnt	IVW	83	-0.0858	0.3500	8.06E-01
Testosterone	ukb-d-30850_irnt	MR Egger	83	0.0010	0.6083	9.99E-01
Testosterone	ukb-d-30850_irnt	WM	83	-0.1562	0.3293	6.35E-01
Total bilirubin	ukb-d-30840_irnt	IVW	113	0.0181	0.0739	8.07E-01
Total bilirubin	ukb-d-30840_irnt	MR Egger	113	0.0168	0.0796	8.34E-01
Total bilirubin	ukb-d-30840_irnt	WM	113	0.0415	0.0329	2.07E-01
Total cholesterol	ieu-a-301	IVW	83	-0.1406	0.1422	3.23E-01
Total cholesterol	ieu-a-301	MR Egger	83	-0.0971	0.2325	6.77E-01
Total cholesterol	ieu-a-301	WM	83	0.0099	0.0869	9.09E-01
Total protein	ukb-d-30860_irnt	IVW	193	-0.1855	0.1632	2.56E-01
Total protein	ukb-d-30860_irnt	MR Egger	193	-0.8579	0.3485	1.47E-02
Total protein	ukb-d-30860_irnt	WM	193	-0.0892	0.1438	5.35E-01
Townsend deprivation index at recruitment	ukb-a-44	IVW	5	0.0515	0.7411	9.45E-01
Townsend deprivation index at recruitment	ukb-a-44	MR Egger	5	1.9492	3.4830	6.15E-01
Townsend deprivation index at recruitment	ukb-a-44	WM	5	0.0493	0.9287	9.58E-01
Transferrin	ieu-a-1052	IVW	8	0.2575	0.7117	7.18E-01
Transferrin	ieu-a-1052	MR Egger	8	-0.3418	1.1065	7.68E-01
Transferrin	ieu-a-1052	WM	8	-0.0148	0.0679	8.27E-01
Triglycerides	ukb-d-30870_irnt	IVW	192	0.1173	0.0826	1.56E-01
Triglycerides	ukb-d-30870_irnt	MR Egger	192	0.0353	0.1201	7.69E-01
Triglycerides	ukb-d-30870_irnt	WM	192	0.0089	0.1037	9.32E-01
Trunk fat mass	ukb-a-291	IVW	265	0.1777	0.1079	9.95E-02
Trunk fat mass	ukb-a-291	MR Egger	265	0.5823	0.3299	7.87E-02
Trunk fat mass	ukb-a-291	WM	265	0.1923	0.1258	1.26E-01
Trunk fat percentage	ukb-a-290	IVW	223	0.2263	0.1393	1.04E-01
Trunk fat percentage	ukb-a-290	MR Egger	223	0.0965	0.5053	8.49E-01
Trunk fat percentage	ukb-a-290	WM	223	0.0642	0.1473	6.63E-01

Trunk fat-free mass	ukb-a-292	IVW	384	-0.2317	0.1192	5.19E-02
Trunk fat-free mass	ukb-a-292	MR Egger	384	0.0408	0.2963	8.90E-01
Trunk fat-free mass	ukb-a-292	WM	384	0.0917	0.1482	5.36E-01
Trunk predicted mass	ukb-a-293	IVW	386	-0.2203	0.1186	6.32E-02
Trunk predicted mass	ukb-a-293	MR Egger	386	0.0252	0.2945	9.32E-01
Trunk predicted mass	ukb-a-293	WM	386	0.0718	0.1493	6.30E-01
Urate	ukb-d-30880_irnt	IVW	205	0.1678	0.0873	5.46E-02
Urate	ukb-d-30880_irnt	MR Egger	205	0.2004	0.1212	9.99E-02
Urate	ukb-d-30880_irnt	WM	205	0.0010	0.0991	9.92E-01
Urea	ukb-d-30670_irnt	IVW	129	-0.1798	0.1812	3.21E-01
Urea	ukb-d-30670_irnt	MR Egger	129	0.2551	0.4241	5.49E-01
Urea	ukb-d-30670_irnt	WM	129	0.0347	0.1679	8.36E-01
Urinary sodium-potassium ratio	ieu-b-72	IVW	23	0.8680	1.0216	3.95E-01
Urinary sodium-potassium ratio	ieu-b-72	MR Egger	23	-8.3754	5.7560	1.60E-01
Urinary sodium-potassium ratio	ieu-b-72	WM	23	-0.0130	0.4777	9.78E-01
Usual walking pace	ukb-a-513	IVW	28	0.2669	0.5780	6.44E-01
Usual walking pace	ukb-a-513	MR Egger	28	3.1390	3.5034	3.78E-01
Usual walking pace	ukb-a-513	WM	28	0.0389	0.6949	9.55E-01
Vitamin D	ukb-d-30890_irnt	IVW	55	-0.0085	0.0909	9.25E-01
Vitamin D	ukb-d-30890_irnt	MR Egger	55	-0.0393	0.1256	7.55E-01
Vitamin D	ukb-d-30890_irnt	WM	55	-0.0353	0.1138	7.57E-01
Waist circumference	ukb-a-382	IVW	214	0.0680	0.1368	6.19E-01
Waist circumference	ukb-a-382	MR Egger	214	0.2336	0.4233	5.82E-01
Waist circumference	ukb-a-382	WM	214	-0.0227	0.1669	8.92E-01
Waist-to-hip ratio	ieu-a-72	IVW	29	-0.2224	0.2814	4.29E-01
Waist-to-hip ratio	ieu-a-72	MR Egger	29	-0.4808	1.2977	7.14E-01
Waist-to-hip ratio	ieu-a-72	WM	29	-0.6143	0.3058	4.46E-02
Weight	ukb-a-249	IVW	319	-0.0313	0.1060	7.68E-01
Weight	ukb-a-249	MR Egger	319	0.4735	0.2779	8.94E-02
Weight	ukb-a-249	WM	319	0.0672	0.1288	6.02E-01
White blood cell (leukocyte) count	ukb-d-30000_irnt	IVW	274	-0.4079	0.1297	1.66E-03
White blood cell (leukocyte) count	ukb-d-30000_irnt	MR Egger	274	-0.9055	0.2832	1.55E-03
White blood cell (leukocyte) count	ukb-d-30000_irnt	WM	274	-0.0646	0.1237	6.01E-01
Whole body fat mass	ukb-a-265	IVW	262	0.1078	0.1072	3.15E-01
Whole body fat mass	ukb-a-265	MR Egger	262	0.5174	0.3245	1.12E-01
Whole body fat mass	ukb-a-265	WM	262	0.0480	0.1270	7.06E-01
Whole body fat-free mass	ukb-a-266	IVW	380	-0.2812	0.1391	4.32E-02
Whole body fat-free mass	ukb-a-266	MR Egger	380	-0.2822	0.3412	4.09E-01
Whole body fat-free mass	ukb-a-266	WM	380	0.0006	0.1501	9.97E-01
Whole body water mass	ukb-a-267	IVW	376	-0.0845	0.1801	6.39E-01
Whole body water mass	ukb-a-267	MR Egger	376	-0.1614	0.4381	7.13E-01
Whole body water mass	ukb-a-267	WM	376	-0.0023	0.1522	9.88E-01
