# Supplementary material for: Assessment of common risk factors of diabetes and chronic kidney disease: a Mendelian randomization study
Source: Front Endocrinol (Lausanne). 2023 Sep 13;14:1265719. doi: 10.3389/fendo.2023.1265719 (PMC10535100; doi:10.3389/fendo.2023.1265719)
Supplement: Supplementary file 2 [file Table_2.pdf]

Supplementary Table 2. MR analyses of the effect of exposures on T2D

| Trait | Trait ID | Method | nsnp | Beta | SE | P |
|---|---|---|---|---|---|---|
| Adiponectin | ieu-a-1 | IVW | 14 | -0.0407 | 0.0588 | 4.88E-01 |
| Adiponectin | ieu-a-1 | MR Egger | 14 | 0.0685 | 0.0713 | 3.56E-01 |
| Adiponectin | ieu-a-1 | WM | 14 | 0.0334 | 0.0400 | 4.04E-01 |
| Alanine aminotransferase | ukb-d-30620_irnt | IVW | 166 | 0.4769 | 0.0856 | 2.55E-08 |
| Alanine aminotransferase | ukb-d-30620_irnt | MR Egger | 166 | 0.0832 | 0.1548 | 5.92E-01 |
| Alanine aminotransferase | ukb-d-30620_irnt | WM | 166 | 0.1769 | 0.0556 | 1.48E-03 |
| Albumin | ukb-d-30600_irnt | IVW | 171 | 0.1756 | 0.0885 | 4.73E-02 |
| Albumin | ukb-d-30600_irnt | MR Egger | 171 | -0.1955 | 0.1728 | 2.60E-01 |
| Albumin | ukb-d-30600_irnt | WM | 171 | -0.0732 | 0.0513 | 1.54E-01 |
| Alcohol intake frequency | ukb-a-25 | IVW | 40 | 0.3373 | 0.1729 | 5.11E-02 |
| Alcohol intake frequency | ukb-a-25 | MR Egger | 40 | -1.3348 | 0.6103 | 3.50E-02 |
| Alcohol intake frequency | ukb-a-25 | WM | 40 | 0.3238 | 0.0918 | 4.22E-04 |
| Alcohol intake versus 10 years previously | ukb-a-32 | IVW | 7 | -0.7001 | 0.9069 | 4.40E-01 |
| Alcohol intake versus 10 years previously | ukb-a-32 | MR Egger | 7 | -11.1031 | 10.4674 | 3.37E-01 |
| Alcohol intake versus 10 years previously | ukb-a-32 | WM | 7 | -0.2159 | 0.4412 | 6.25E-01 |
| Alcoholic drinks per week | ieu-b-73 | IVW | 32 | 0.1233 | 0.2353 | 6.00E-01 |
| Alcoholic drinks per week | ieu-b-73 | MR Egger | 32 | 1.6241 | 0.6718 | 2.19E-02 |
| Alcoholic drinks per week | ieu-b-73 | WM | 32 | 0.1109 | 0.1888 | 5.57E-01 |
| Alkaline phosphatase | ukb-d-30610_irnt | IVW | 261 | 0.0044 | 0.0326 | 8.93E-01 |
| Alkaline phosphatase | ukb-d-30610_irnt | MR Egger | 261 | -0.0284 | 0.0542 | 6.01E-01 |
| Alkaline phosphatase | ukb-d-30610_irnt | WM | 261 | 0.0198 | 0.0382 | 6.04E-01 |
| Apoliprotein A | ukb-d-30630_irnt | IVW | 198 | -0.1790 | 0.0415 | 1.64E-05 |
| Apoliprotein A | ukb-d-30630_irnt | MR Egger | 198 | -0.0415 | 0.0624 | 5.07E-01 |
| Apoliprotein A | ukb-d-30630_irnt | WM | 198 | -0.1303 | 0.0362 | 3.18E-04 |
| Apoliprotein B | ukb-d-30640_irnt | IVW | 132 | 0.0014 | 0.0462 | 9.76E-01 |
| Apoliprotein B | ukb-d-30640_irnt | MR Egger | 132 | -0.0929 | 0.0660 | 1.61E-01 |
| Apoliprotein B | ukb-d-30640_irnt | WM | 132 | -0.0435 | 0.0323 | 1.77E-01 |
| Aspartate aminotransferase | ukb-d-30650_irnt | IVW | 197 | 0.0229 | 0.0478 | 6.32E-01 |
| Aspartate aminotransferase | ukb-d-30650_irnt | MR Egger | 197 | -0.0740 | 0.0891 | 4.07E-01 |
| Aspartate aminotransferase | ukb-d-30650_irnt | WM | 197 | -0.0399 | 0.0442 | 3.67E-01 |
| Basal metabolic rate | ukb-a-268 | IVW | 355 | 0.2903 | 0.0739 | 8.48E-05 |
| Basal metabolic rate | ukb-a-268 | MR Egger | 355 | -0.3124 | 0.1813 | 8.58E-02 |
| Basal metabolic rate | ukb-a-268 | WM | 355 | 0.1472 | 0.0620 | 1.77E-02 |
| Basophill percentage | ukb-d-30220_irnt | IVW | 93 | 0.0014 | 0.0510 | 9.78E-01 |
| Basophill percentage | ukb-d-30220_irnt | MR Egger | 93 | 0.0927 | 0.0958 | 3.36E-01 |
| Basophill percentage | ukb-d-30220_irnt | WM | 93 | 0.0239 | 0.0502 | 6.34E-01 |
| Birth weight | ukb-a-198 | IVW | 75 | -0.4428 | 0.1271 | 4.92E-04 |
| Birth weight | ukb-a-198 | MR Egger | 75 | -1.5905 | 0.4299 | 4.15E-04 |
| Birth weight | ukb-a-198 | WM | 75 | -0.2920 | 0.0728 | 6.03E-05 |
| Birth weight of first child | ukb-a-318 | IVW | 41 | 0.0976 | 0.1192 | 4.13E-01 |
| Birth weight of first child | ukb-a-318 | MR Egger | 41 | 0.6025 | 0.4343 | 1.73E-01 |
| Birth weight of first child | ukb-a-318 | WM | 41 | -0.0626 | 0.0671 | 3.52E-01 |

| | | | | | | |
|---|---|---|---|---|---|---|
| Body fat | ieu-a-999 | IVW | 10 | 0.8641 | 0.4753 | 6.91E-02 |
| Body fat | ieu-a-999 | MR Egger | 10 | 4.6192 | 1.8503 | 3.71E-02 |
| Body fat | ieu-a-999 | WM | 10 | 1.1898 | 0.1686 | 1.71E-12 |
| Body fat percentage | ukb-a-264 | IVW | 238 | 0.7731 | 0.0916 | 3.20E-17 |
| Body fat percentage | ukb-a-264 | MR Egger | 238 | 1.3457 | 0.3347 | 7.83E-05 |
| Body fat percentage | ukb-a-264 | WM | 238 | 0.8731 | 0.0674 | 2.42E-38 |
| body mass index | ieu-b-40 | IVW | 476 | 0.8495 | 0.0514 | 2.69E-61 |
| body mass index | ieu-b-40 | MR Egger | 476 | 1.0407 | 0.1358 | 1.03E-13 |
| body mass index | ieu-b-40 | WM | 476 | 0.9128 | 0.0442 | 1.28E-94 |
| Calcium | ukb-d-30680_irnt | IVW | 172 | 0.0034 | 0.0500 | 9.45E-01 |
| Calcium | ukb-d-30680_irnt | MR Egger | 172 | -0.1514 | 0.0915 | 9.98E-02 |
| Calcium | ukb-d-30680_irnt | WM | 172 | 0.0018 | 0.0402 | 9.65E-01 |
| Cholesterol | ukb-d-30690_irnt | IVW | 139 | -0.0760 | 0.0513 | 1.39E-01 |
| Cholesterol | ukb-d-30690_irnt | MR Egger | 139 | -0.1310 | 0.0838 | 1.20E-01 |
| Cholesterol | ukb-d-30690_irnt | WM | 139 | -0.0375 | 0.0402 | 3.51E-01 |
| Cigarettes per Day | ieu-b-25 | IVW | 22 | -0.0041 | 0.0472 | 9.30E-01 |
| Cigarettes per Day | ieu-b-25 | MR Egger | 22 | -0.1091 | 0.0797 | 1.86E-01 |
| Cigarettes per Day | ieu-b-25 | WM | 22 | -0.0855 | 0.0430 | 4.69E-02 |
| C-reactive protein | ukb-d-30710_irnt | IVW | 170 | 0.0267 | 0.0452 | 5.55E-01 |
| C-reactive protein | ukb-d-30710_irnt | MR Egger | 170 | -0.0214 | 0.0623 | 7.32E-01 |
| C-reactive protein | ukb-d-30710_irnt | WM | 170 | 0.0225 | 0.0353 | 5.24E-01 |
| Creatinine | ukb-d-30700_irnt | IVW | 268 | -0.0794 | 0.0464 | 8.73E-02 |
| Creatinine | ukb-d-30700_irnt | MR Egger | 268 | -0.0892 | 0.1076 | 4.08E-01 |
| Creatinine | ukb-d-30700_irnt | WM | 268 | -0.1523 | 0.0474 | 1.31E-03 |
| Creatinine (enzymatic) in urine | ukb-a-333 | IVW | 21 | -0.2065 | 0.2298 | 3.69E-01 |
| Creatinine (enzymatic) in urine | ukb-a-333 | MR Egger | 21 | -0.9516 | 0.9621 | 3.35E-01 |
| Creatinine (enzymatic) in urine | ukb-a-333 | WM | 21 | -0.3108 | 0.1859 | 9.45E-02 |
| Current tobacco smoking | ukb-a-16 | IVW | 16 | -0.3001 | 0.3211 | 3.50E-01 |
| Current tobacco smoking | ukb-a-16 | MR Egger | 16 | -2.9622 | 1.3923 | 5.16E-02 |
| Current tobacco smoking | ukb-a-16 | WM | 16 | -0.4221 | 0.3362 | 2.09E-01 |
| Cystatin C | ukb-d-30720_irnt | IVW | 261 | 0.0595 | 0.0371 | 1.09E-01 |
| Cystatin C | ukb-d-30720_irnt | MR Egger | 261 | -0.0245 | 0.0507 | 6.29E-01 |
| Cystatin C | ukb-d-30720_irnt | WM | 261 | -0.0024 | 0.0226 | 9.17E-01 |
| Daytime dozing / sleeping (narcolepsy) | ukb-a-15 | IVW | 19 | 0.1796 | 0.5441 | 7.41E-01 |
| Daytime dozing / sleeping (narcolepsy) | ukb-a-15 | MR Egger | 19 | 3.0657 | 2.6365 | 2.61E-01 |
| Daytime dozing / sleeping (narcolepsy) | ukb-a-15 | WM | 19 | -0.0420 | 0.4016 | 9.17E-01 |
| Diastolic blood pressure | ukb-a-359 | IVW | 165 | 0.2257 | 0.0609 | 2.12E-04 |
| Diastolic blood pressure | ukb-a-359 | MR Egger | 165 | 0.1264 | 0.2141 | 5.56E-01 |
| Diastolic blood pressure | ukb-a-359 | WM | 165 | 0.1738 | 0.0570 | 2.31E-03 |
| Direct bilirubin | ukb-d-30660_irnt | IVW | 63 | 0.0053 | 0.0284 | 8.53E-01 |
| Direct bilirubin | ukb-d-30660_irnt | MR Egger | 63 | 0.0211 | 0.0311 | 5.00E-01 |
| Direct bilirubin | ukb-d-30660_irnt | WM | 63 | 0.0195 | 0.0125 | 1.19E-01 |
| Drive faster than motorway speed limit | ukb-a-8 | IVW | 13 | -0.4592 | 0.3909 | 2.40E-01 |
| Drive faster than motorway speed limit | ukb-a-8 | MR Egger | 13 | 1.3446 | 2.0985 | 5.35E-01 |
| Drive faster than motorway speed limit | ukb-a-8 | WM | 13 | -0.1578 | 0.2796 | 5.72E-01 |

| | | | | | | |
|---|---|---|---|---|---|---|
| Eosinophill percentage | ukb-d-30210_irnt | IVW | 266 | 0.0038 | 0.0288 | 8.94E-01 |
| Eosinophill percentage | ukb-d-30210_irnt | MR Egger | 266 | -0.0302 | 0.0592 | 6.10E-01 |
| Eosinophill percentage | ukb-d-30210_irnt | WM | 266 | -0.0088 | 0.0330 | 7.91E-01 |
| Fasting glucose | ieu-b-114 | IVW | 30 | 1.7376 | 0.3797 | 4.74E-06 |
| Fasting glucose | ieu-b-114 | MR Egger | 30 | -0.1693 | 0.7181 | 8.15E-01 |
| Fasting glucose | ieu-b-114 | WM | 30 | 1.0717 | 0.1560 | 6.51E-12 |
| Fasting insulin | ieu-b-116 | IVW | 14 | 2.2018 | 0.4181 | 1.39E-07 |
| Fasting insulin | ieu-b-116 | MR Egger | 14 | 5.6558 | 2.0105 | 1.57E-02 |
| Fasting insulin | ieu-b-116 | WM | 14 | 2.0706 | 0.2928 | 1.53E-12 |
| Fluid intelligence score | ukb-a-196 | IVW | 42 | -0.0614 | 0.0426 | 1.50E-01 |
| Fluid intelligence score | ukb-a-196 | MR Egger | 42 | -0.1455 | 0.1980 | 4.67E-01 |
| Fluid intelligence score | ukb-a-196 | WM | 42 | -0.0648 | 0.0339 | 5.61E-02 |
| Forced expiratory volume in 1-second (FEV1) | ukb-a-337 | IVW | 144 | -0.3789 | 0.1012 | 1.81E-04 |
| Forced expiratory volume in 1-second (FEV1) | ukb-a-337 | MR Egger | 144 | -0.9019 | 0.3354 | 8.03E-03 |
| Forced expiratory volume in 1-second (FEV1) | ukb-a-337 | WM | 144 | -0.2786 | 0.0725 | 1.22E-04 |
| Forced vital capacity (FVC) | ukb-a-336 | IVW | 202 | -0.3988 | 0.0798 | 5.89E-07 |
| Forced vital capacity (FVC) | ukb-a-336 | MR Egger | 202 | -0.7808 | 0.2317 | 9.04E-04 |
| Forced vital capacity (FVC) | ukb-a-336 | WM | 202 | -0.3487 | 0.0605 | 8.18E-09 |
| Gamma glutamyltransferase | ukb-d-30730_irnt | IVW | 227 | 0.0682 | 0.0348 | 5.03E-02 |
| Gamma glutamyltransferase | ukb-d-30730_irnt | MR Egger | 227 | -0.0939 | 0.0543 | 8.47E-02 |
| Gamma glutamyltransferase | ukb-d-30730_irnt | WM | 227 | 0.0663 | 0.0356 | 6.26E-02 |
| Getting up in morning | ukb-a-10 | IVW | 36 | 0.4543 | 0.3171 | 1.52E-01 |
| Getting up in morning | ukb-a-10 | MR Egger | 36 | -0.0651 | 1.1517 | 9.55E-01 |
| Getting up in morning | ukb-a-10 | WM | 36 | 0.1135 | 0.1888 | 5.48E-01 |
| Glucose | ukb-d-30740_irnt | IVW | 86 | 1.0948 | 0.1363 | 9.51E-16 |
| Glucose | ukb-d-30740_irnt | MR Egger | 86 | 0.4796 | 0.2060 | 2.23E-02 |
| Glucose | ukb-d-30740_irnt | WM | 86 | 0.5864 | 0.0916 | 1.52E-10 |
| Glycated haemoglobin | ukb-d-30750_irnt | IVW | 249 | 0.7459 | 0.0632 | 3.91E-32 |
| Glycated haemoglobin | ukb-d-30750_irnt | MR Egger | 249 | 0.5305 | 0.1059 | 1.03E-06 |
| Glycated haemoglobin | ukb-d-30750_irnt | WM | 249 | 0.2119 | 0.0382 | 2.85E-08 |
| Haematocrit percentage | ukb-d-30030_irnt | IVW | 211 | 0.0839 | 0.0552 | 1.28E-01 |
| Haematocrit percentage | ukb-d-30030_irnt | MR Egger | 211 | -0.1031 | 0.1168 | 3.78E-01 |
| Haematocrit percentage | ukb-d-30030_irnt | WM | 211 | 0.0620 | 0.0528 | 2.40E-01 |
| Haemoglobin concentration | ukb-d-30020_irnt | IVW | 232 | 0.1079 | 0.0557 | 5.26E-02 |
| Haemoglobin concentration | ukb-d-30020_irnt | MR Egger | 232 | -0.0692 | 0.1137 | 5.43E-01 |
| Haemoglobin concentration | ukb-d-30020_irnt | WM | 232 | 0.0466 | 0.0506 | 3.57E-01 |
| HDL cholesterol | ukb-d-30760_irnt | IVW | 222 | -0.2713 | 0.0407 | 2.48E-11 |
| HDL cholesterol | ukb-d-30760_irnt | MR Egger | 222 | -0.0547 | 0.0567 | 3.36E-01 |
| HDL cholesterol | ukb-d-30760_irnt | WM | 222 | -0.1016 | 0.0296 | 6.02E-04 |
| Heart rate | ieu-a-1056 | IVW | 14 | 0.0062 | 0.0103 | 5.48E-01 |
| Heart rate | ieu-a-1056 | MR Egger | 14 | 0.1007 | 0.0312 | 7.27E-03 |
| Heart rate | ieu-a-1056 | WM | 14 | 0.0111 | 0.0093 | 2.33E-01 |
| Heel bone mineral density (BMD) T-score | ukb-a-500 | IVW | 232 | 0.0363 | 0.0254 | 1.53E-01 |
| Heel bone mineral density (BMD) T-score | ukb-a-500 | MR Egger | 232 | 0.0129 | 0.0490 | 7.93E-01 |
| Heel bone mineral density (BMD) T-score | ukb-a-500 | WM | 232 | 0.0074 | 0.0266 | 7.80E-01 |

| | | | | | | |
|---|---|---|---|---|---|---|
| High light scatter reticulocyte count | ukb-d-30300_irnt | IVW | 250 | 0.0846 | 0.0414 | 4.10E-02 |
| High light scatter reticulocyte count | ukb-d-30300_irnt | MR Egger | 250 | -0.1483 | 0.0788 | 6.11E-02 |
| High light scatter reticulocyte count | ukb-d-30300_irnt | WM | 250 | 0.0069 | 0.0364 | 8.49E-01 |
| High light scatter reticulocyte percentage | ukb-d-30290_irnt | IVW | 260 | 0.0672 | 0.0397 | 9.06E-02 |
| High light scatter reticulocyte percentage | ukb-d-30290_irnt | MR Egger | 260 | -0.1422 | 0.0743 | 5.67E-02 |
| High light scatter reticulocyte percentage | ukb-d-30290_irnt | WM | 260 | -0.0125 | 0.0345 | 7.17E-01 |
| Hip circumference | ukb-a-388 | IVW | 270 | 0.3067 | 0.0754 | 4.72E-05 |
| Hip circumference | ukb-a-388 | MR Egger | 270 | 0.1370 | 0.2159 | 5.26E-01 |
| Hip circumference | ukb-a-388 | WM | 270 | 0.3802 | 0.0562 | 1.33E-11 |
| IGF-1 | ukb-d-30770_irnt | IVW | 288 | 0.1057 | 0.0359 | 3.25E-03 |
| IGF-1 | ukb-d-30770_irnt | MR Egger | 288 | 0.1523 | 0.0701 | 3.07E-02 |
| IGF-1 | ukb-d-30770_irnt | WM | 288 | 0.0154 | 0.0398 | 6.98E-01 |
| Immature reticulocyte fraction | ukb-d-30280_irnt | IVW | 181 | 0.1260 | 0.0441 | 4.22E-03 |
| Immature reticulocyte fraction | ukb-d-30280_irnt | MR Egger | 181 | 0.0327 | 0.0801 | 6.84E-01 |
| Immature reticulocyte fraction | ukb-d-30280_irnt | WM | 181 | 0.0826 | 0.0420 | 4.95E-02 |
| Impedance of whole body | ukb-a-269 | IVW | 333 | -0.2794 | 0.0730 | 1.29E-04 |
| Impedance of whole body | ukb-a-269 | MR Egger | 333 | -0.4529 | 0.2049 | 2.78E-02 |
| Impedance of whole body | ukb-a-269 | WM | 333 | -0.1347 | 0.0542 | 1.30E-02 |
| Job involves mainly walking or standing | ukb-a-502 | IVW | 7 | 0.2193 | 0.1973 | 2.66E-01 |
| Job involves mainly walking or standing | ukb-a-502 | MR Egger | 7 | 1.2719 | 2.1219 | 5.75E-01 |
| Job involves mainly walking or standing | ukb-a-502 | WM | 7 | 0.2130 | 0.1759 | 2.26E-01 |
| LDL direct | ukb-d-30780_irnt | IVW | 128 | -0.0225 | 0.0470 | 6.32E-01 |
| LDL direct | ukb-d-30780_irnt | MR Egger | 128 | -0.1091 | 0.0700 | 1.22E-01 |
| LDL direct | ukb-d-30780_irnt | WM | 128 | -0.0389 | 0.0386 | 3.13E-01 |
| Length of menstrual cycle | ukb-a-351 | IVW | 6 | -0.0332 | 0.0897 | 7.11E-01 |
| Length of menstrual cycle | ukb-a-351 | MR Egger | 6 | -0.1018 | 0.2209 | 6.69E-01 |
| Length of menstrual cycle | ukb-a-351 | WM | 6 | -0.0618 | 0.0603 | 3.06E-01 |
| Lipoprotein A | ukb-d-30790_irnt | IVW | 17 | 0.0192 | 0.0139 | 1.68E-01 |
| Lipoprotein A | ukb-d-30790_irnt | MR Egger | 17 | 0.0096 | 0.0172 | 5.83E-01 |
| Lipoprotein A | ukb-d-30790_irnt | WM | 17 | 0.0226 | 0.0112 | 4.40E-02 |
| Lymphocyte count | ukb-d-30120_irnt | IVW | 279 | 0.0557 | 0.0336 | 9.76E-02 |
| Lymphocyte count | ukb-d-30120_irnt | MR Egger | 279 | -0.0518 | 0.0800 | 5.18E-01 |
| Lymphocyte count | ukb-d-30120_irnt | WM | 279 | 0.0725 | 0.0389 | 6.26E-02 |
| Lymphocyte percentage | ukb-d-30180_irnt | IVW | 238 | 0.0209 | 0.0353 | 5.54E-01 |
| Lymphocyte percentage | ukb-d-30180_irnt | MR Egger | 238 | 0.0108 | 0.0786 | 8.91E-01 |
| Lymphocyte percentage | ukb-d-30180_irnt | WM | 238 | 0.0220 | 0.0390 | 5.72E-01 |
| Mean corpuscular haemoglobin | ukb-d-30050_irnt | IVW | 295 | -0.0234 | 0.0210 | 2.65E-01 |
| Mean corpuscular haemoglobin | ukb-d-30050_irnt | MR Egger | 295 | 0.0379 | 0.0337 | 2.61E-01 |
| Mean corpuscular haemoglobin | ukb-d-30050_irnt | WM | 295 | -0.0150 | 0.0239 | 5.30E-01 |
| Mean corpuscular haemoglobin concentration | ukb-d-30060_irnt | IVW | 84 | -0.0621 | 0.0556 | 2.64E-01 |
| Mean corpuscular haemoglobin concentration | ukb-d-30060_irnt | MR Egger | 84 | -0.0298 | 0.1102 | 7.88E-01 |
| Mean corpuscular haemoglobin concentration | ukb-d-30060_irnt | WM | 84 | -0.0590 | 0.0541 | 2.76E-01 |
| Mean corpuscular volume | ukb-d-30040_irnt | IVW | 301 | -0.0097 | 0.0219 | 6.56E-01 |
| Mean corpuscular volume | ukb-d-30040_irnt | MR Egger | 301 | 0.0327 | 0.0373 | 3.80E-01 |
| Mean corpuscular volume | ukb-d-30040_irnt | WM | 301 | -0.0057 | 0.0255 | 8.22E-01 |

| | | | | | | |
|---|---|---|---|---|---|---|
| Mean platelet (thrombocyte) volume | ukb-d-30100_irnt | IVW | 356 | 0.0050 | 0.0167 | 7.67E-01 |
| Mean platelet (thrombocyte) volume | ukb-d-30100_irnt | MR Egger | 356 | -0.0133 | 0.0250 | 5.96E-01 |
| Mean platelet (thrombocyte) volume | ukb-d-30100_irnt | WM | 356 | 0.0053 | 0.0207 | 7.98E-01 |
| Mean reticulocyte volume | ukb-d-30260_irnt | IVW | 268 | 0.0022 | 0.0302 | 9.41E-01 |
| Mean reticulocyte volume | ukb-d-30260_irnt | MR Egger | 268 | 0.0993 | 0.0543 | 6.83E-02 |
| Mean reticulocyte volume | ukb-d-30260_irnt | WM | 268 | 0.0378 | 0.0273 | 1.65E-01 |
| Mean sphered cell volume | ukb-d-30270_irnt | IVW | 276 | 0.0033 | 0.0333 | 9.21E-01 |
| Mean sphered cell volume | ukb-d-30270_irnt | MR Egger | 276 | 0.1848 | 0.0572 | 1.38E-03 |
| Mean sphered cell volume | ukb-d-30270_irnt | WM | 276 | 0.0851 | 0.0294 | 3.79E-03 |
| Mean time to correctly identify matches | ukb-a-199 | IVW | 26 | 0.0267 | 0.1606 | 8.68E-01 |
| Mean time to correctly identify matches | ukb-a-199 | MR Egger | 26 | -0.3479 | 1.0703 | 7.48E-01 |
| Mean time to correctly identify matches | ukb-a-199 | WM | 26 | 0.1173 | 0.1573 | 4.56E-01 |
| Microalbumin in urine | ukb-d-30500_irnt | IVW | 4 | 0.0871 | 0.2488 | 7.26E-01 |
| Microalbumin in urine | ukb-d-30500_irnt | MR Egger | 4 | -0.1730 | 0.5548 | 7.85E-01 |
| Microalbumin in urine | ukb-d-30500_irnt | WM | 4 | 0.2040 | 0.1920 | 2.88E-01 |
| Monocyte count | ukb-d-30130_irnt | IVW | 277 | -0.0422 | 0.0256 | 9.92E-02 |
| Monocyte count | ukb-d-30130_irnt | MR Egger | 277 | -0.0837 | 0.0416 | 4.49E-02 |
| Monocyte count | ukb-d-30130_irnt | WM | 277 | -0.0330 | 0.0314 | 2.94E-01 |
| Monocyte percentage | ukb-d-30190_irnt | IVW | 257 | -0.0350 | 0.0273 | 2.00E-01 |
| Monocyte percentage | ukb-d-30190_irnt | MR Egger | 257 | -0.0684 | 0.0455 | 1.34E-01 |
| Monocyte percentage | ukb-d-30190_irnt | WM | 257 | -0.0433 | 0.0301 | 1.50E-01 |
| Morning/evening person (chronotype) | ukb-a-11 | IVW | 80 | -0.1958 | 0.1078 | 6.93E-02 |
| Morning/evening person (chronotype) | ukb-a-11 | MR Egger | 80 | -0.1714 | 0.2522 | 4.99E-01 |
| Morning/evening person (chronotype) | ukb-a-11 | WM | 80 | -0.0213 | 0.0915 | 8.16E-01 |
| Nap during day | ukb-a-12 | IVW | 47 | 0.4593 | 0.1821 | 1.17E-02 |
| Nap during day | ukb-a-12 | MR Egger | 47 | 1.4330 | 0.6808 | 4.09E-02 |
| Nap during day | ukb-a-12 | WM | 47 | 0.1798 | 0.1924 | 3.50E-01 |
| Neuroticism | ieu-a-1007 | IVW | 9 | -0.0960 | 0.2880 | 7.39E-01 |
| Neuroticism | ieu-a-1007 | MR Egger | 9 | -1.2039 | 2.4178 | 6.34E-01 |
| Neuroticism | ieu-a-1007 | WM | 9 | -0.1409 | 0.2188 | 5.19E-01 |
| Neuroticism score | ukb-a-230 | IVW | 62 | 0.0311 | 0.0359 | 3.86E-01 |
| Neuroticism score | ukb-a-230 | MR Egger | 62 | -0.0001 | 0.2125 | 9.99E-01 |
| Neuroticism score | ukb-a-230 | WM | 62 | 0.0200 | 0.0312 | 5.22E-01 |
| Neutrophill count | ukb-d-30140_irnt | IVW | 239 | -0.0382 | 0.0437 | 3.82E-01 |
| Neutrophill count | ukb-d-30140_irnt | MR Egger | 239 | -0.1573 | 0.0925 | 9.02E-02 |
| Neutrophill count | ukb-d-30140_irnt | WM | 239 | 0.0019 | 0.0416 | 9.64E-01 |
| Neutrophill percentage | ukb-d-30200_irnt | IVW | 238 | -0.0550 | 0.0360 | 1.27E-01 |
| Neutrophill percentage | ukb-d-30200_irnt | MR Egger | 238 | -0.0224 | 0.0816 | 7.84E-01 |
| Neutrophill percentage | ukb-d-30200_irnt | WM | 238 | -0.0178 | 0.0394 | 6.51E-01 |
| Overall health rating | ukb-a-251 | IVW | 49 | 0.8246 | 0.1549 | 1.02E-07 |
| Overall health rating | ukb-a-251 | MR Egger | 49 | 0.9808 | 0.9246 | 2.94E-01 |
| Overall health rating | ukb-a-251 | WM | 49 | 0.6440 | 0.1652 | 9.67E-05 |
| Past tobacco smoking | ukb-a-17 | IVW | 40 | -0.0348 | 0.1044 | 7.39E-01 |
| Past tobacco smoking | ukb-a-17 | MR Egger | 40 | 0.1499 | 0.4228 | 7.25E-01 |
| Past tobacco smoking | ukb-a-17 | WM | 40 | 0.0775 | 0.1000 | 4.38E-01 |

| | | | | | | |
|---|---|---|---|---|---|---|
| Peak expiratory flow (PEF) | ukb-a-338 | IVW | 78 | -0.4699 | 0.1749 | 7.21E-03 |
| Peak expiratory flow (PEF) | ukb-a-338 | MR Egger | 78 | -1.1813 | 0.6364 | 6.73E-02 |
| Peak expiratory flow (PEF) | ukb-a-338 | WM | 78 | -0.1681 | 0.0976 | 8.48E-02 |
| Phosphate | ukb-d-30810_irnt | IVW | 134 | 0.0145 | 0.0504 | 7.74E-01 |
| Phosphate | ukb-d-30810_irnt | MR Egger | 134 | -0.0292 | 0.0834 | 7.27E-01 |
| Phosphate | ukb-d-30810_irnt | WM | 134 | -0.0611 | 0.0454 | 1.78E-01 |
| Platelet count | ukb-d-30080_irnt | IVW | 350 | -0.0224 | 0.0251 | 3.71E-01 |
| Platelet count | ukb-d-30080_irnt | MR Egger | 350 | -0.0130 | 0.0449 | 7.72E-01 |
| Platelet count | ukb-d-30080_irnt | WM | 350 | 0.0066 | 0.0265 | 8.02E-01 |
| Platelet crit | ukb-d-30090_irnt | IVW | 317 | -0.0367 | 0.0289 | 2.05E-01 |
| Platelet crit | ukb-d-30090_irnt | MR Egger | 317 | 0.0293 | 0.0517 | 5.71E-01 |
| Platelet crit | ukb-d-30090_irnt | WM | 317 | -0.0121 | 0.0294 | 6.81E-01 |
| Platelet distribution width | ukb-d-30110_irnt | IVW | 283 | 0.0452 | 0.0228 | 4.73E-02 |
| Platelet distribution width | ukb-d-30110_irnt | MR Egger | 283 | -0.0186 | 0.0332 | 5.75E-01 |
| Platelet distribution width | ukb-d-30110_irnt | WM | 283 | -0.0234 | 0.0224 | 2.97E-01 |
| Potassium in urine | ukb-a-334 | IVW | 9 | -0.4848 | 0.3229 | 1.33E-01 |
| Potassium in urine | ukb-a-334 | MR Egger | 9 | 1.7185 | 2.2813 | 4.76E-01 |
| Potassium in urine | ukb-a-334 | WM | 9 | -0.1485 | 0.2454 | 5.45E-01 |
| Pulse rate | ukb-a-3 | IVW | 189 | 0.1351 | 0.0487 | 5.57E-03 |
| Pulse rate | ukb-a-3 | MR Egger | 189 | 0.0108 | 0.1149 | 9.25E-01 |
| Pulse rate | ukb-a-3 | WM | 189 | 0.0859 | 0.0432 | 4.67E-02 |
| QRS duration | ukb-d-12340_irnt | IVW | 5 | -0.1166 | 0.0432 | 6.93E-03 |
| QRS duration | ukb-d-12340_irnt | MR Egger | 5 | -0.0879 | 0.2196 | 7.16E-01 |
| QRS duration | ukb-d-12340_irnt | WM | 5 | -0.1504 | 0.0510 | 3.17E-03 |
| Red blood cell (erythrocyte) count | ukb-d-30010_irnt | IVW | 293 | 0.0318 | 0.0348 | 3.61E-01 |
| Red blood cell (erythrocyte) count | ukb-d-30010_irnt | MR Egger | 293 | -0.0886 | 0.0648 | 1.73E-01 |
| Red blood cell (erythrocyte) count | ukb-d-30010_irnt | WM | 293 | 0.0365 | 0.0405 | 3.67E-01 |
| Red blood cell (erythrocyte) distribution width | ukb-d-30070_irnt | IVW | 246 | 0.0476 | 0.0319 | 1.36E-01 |
| Red blood cell (erythrocyte) distribution width | ukb-d-30070_irnt | MR Egger | 246 | -0.0035 | 0.0568 | 9.50E-01 |
| Red blood cell (erythrocyte) distribution width | ukb-d-30070_irnt | WM | 246 | 0.0044 | 0.0294 | 8.82E-01 |
| Reticulocyte count | ukb-d-30250_irnt | IVW | 237 | 0.0475 | 0.0428 | 2.67E-01 |
| Reticulocyte count | ukb-d-30250_irnt | MR Egger | 237 | -0.1943 | 0.0768 | 1.20E-02 |
| Reticulocyte count | ukb-d-30250_irnt | WM | 237 | -0.0811 | 0.0384 | 3.45E-02 |
| Reticulocyte percentage | ukb-d-30240_irnt | IVW | 227 | 0.0323 | 0.0419 | 4.40E-01 |
| Reticulocyte percentage | ukb-d-30240_irnt | MR Egger | 227 | -0.2123 | 0.0760 | 5.65E-03 |
| Reticulocyte percentage | ukb-d-30240_irnt | WM | 227 | -0.1217 | 0.0355 | 6.16E-04 |
| Serum cystatin C (eGFRcys) | ieu-a-1106 | IVW | 5 | 0.1448 | 0.3073 | 6.38E-01 |
| Serum cystatin C (eGFRcys) | ieu-a-1106 | MR Egger | 5 | -0.0320 | 0.5111 | 9.54E-01 |
| Serum cystatin C (eGFRcys) | ieu-a-1106 | WM | 5 | 0.0425 | 0.1245 | 7.33E-01 |
| SHBG | ukb-d-30830_irnt | IVW | 221 | -0.2512 | 0.0511 | 8.97E-07 |
| SHBG | ukb-d-30830_irnt | MR Egger | 221 | -0.0081 | 0.0751 | 9.14E-01 |
| SHBG | ukb-d-30830_irnt | WM | 221 | -0.1612 | 0.0349 | 3.74E-06 |
| Sitting height | ukb-a-195 | IVW | 411 | -0.0702 | 0.0434 | 1.06E-01 |
| Sitting height | ukb-a-195 | MR Egger | 411 | -0.2829 | 0.1079 | 9.06E-03 |
| Sitting height | ukb-a-195 | WM | 411 | -0.0366 | 0.0380 | 3.36E-01 |

| | | | | | | |
|---|---|---|---|---|---|---|
| Sleep duration | ukb-a-9 | IVW | 41 | -0.1387 | 0.2657 | 6.02E-01 |
| Sleep duration | ukb-a-9 | MR Egger | 41 | 0.2643 | 1.0859 | 8.09E-01 |
| Sleep duration | ukb-a-9 | WM | 41 | 0.0279 | 0.1768 | 8.75E-01 |
| Sleeplessness / insomnia | ukb-a-13 | IVW | 28 | 0.4017 | 0.2651 | 1.30E-01 |
| Sleeplessness / insomnia | ukb-a-13 | MR Egger | 28 | 0.5887 | 0.7683 | 4.50E-01 |
| Sleeplessness / insomnia | ukb-a-13 | WM | 28 | 0.5098 | 0.2139 | 1.71E-02 |
| Sodium in urine | ukb-a-335 | IVW | 29 | 0.3615 | 0.3583 | 3.13E-01 |
| Sodium in urine | ukb-a-335 | MR Egger | 29 | -0.6504 | 1.6379 | 6.94E-01 |
| Sodium in urine | ukb-a-335 | WM | 29 | 0.3212 | 0.1546 | 3.77E-02 |
| Standing height | ukb-a-389 | IVW | 564 | -0.1306 | 0.0335 | 9.45E-05 |
| Standing height | ukb-a-389 | MR Egger | 564 | -0.1675 | 0.0733 | 2.26E-02 |
| Standing height | ukb-a-389 | WM | 564 | -0.1052 | 0.0331 | 1.49E-03 |
| Systolic blood pressure | ukb-a-360 | IVW | 145 | 0.3031 | 0.0611 | 7.09E-07 |
| Systolic blood pressure | ukb-a-360 | MR Egger | 145 | 0.1287 | 0.2038 | 5.29E-01 |
| Systolic blood pressure | ukb-a-360 | WM | 145 | 0.2186 | 0.0598 | 2.59E-04 |
| telomere length | ieu-b-4879 | IVW | 129 | 0.0340 | 0.0545 | 5.33E-01 |
| telomere length | ieu-b-4879 | MR Egger | 129 | -0.0118 | 0.0962 | 9.03E-01 |
| telomere length | ieu-b-4879 | WM | 129 | 0.0887 | 0.0539 | 9.98E-02 |
| Testosterone | ukb-d-30850_irnt | IVW | 83 | -0.1733 | 0.1387 | 2.11E-01 |
| Testosterone | ukb-d-30850_irnt | MR Egger | 83 | 0.0181 | 0.2395 | 9.40E-01 |
| Testosterone | ukb-d-30850_irnt | WM | 83 | -0.1999 | 0.1023 | 5.08E-02 |
| Total bilirubin | ukb-d-30840_irnt | IVW | 113 | -0.0005 | 0.0292 | 9.87E-01 |
| Total bilirubin | ukb-d-30840_irnt | MR Egger | 113 | 0.0226 | 0.0309 | 4.66E-01 |
| Total bilirubin | ukb-d-30840_irnt | WM | 113 | 0.0179 | 0.0097 | 6.63E-02 |
| Total cholesterol | ieu-a-301 | IVW | 83 | -0.0381 | 0.0372 | 3.06E-01 |
| Total cholesterol | ieu-a-301 | MR Egger | 83 | -0.0794 | 0.0606 | 1.94E-01 |
| Total cholesterol | ieu-a-301 | WM | 83 | -0.0188 | 0.0320 | 5.58E-01 |
| Total protein | ukb-d-30860_irnt | IVW | 193 | 0.0693 | 0.0623 | 2.66E-01 |
| Total protein | ukb-d-30860_irnt | MR Egger | 193 | -0.1325 | 0.1352 | 3.29E-01 |
| Total protein | ukb-d-30860_irnt | WM | 193 | -0.0744 | 0.0465 | 1.10E-01 |
| Townsend deprivation index at recruitment | ukb-a-44 | IVW | 5 | 0.3647 | 0.2329 | 1.17E-01 |
| Townsend deprivation index at recruitment | ukb-a-44 | MR Egger | 5 | 1.6168 | 1.0897 | 2.35E-01 |
| Townsend deprivation index at recruitment | ukb-a-44 | WM | 5 | 0.3070 | 0.2888 | 2.88E-01 |
| Transferrin | ieu-a-1052 | IVW | 8 | -0.0491 | 0.0359 | 1.71E-01 |
| Transferrin | ieu-a-1052 | MR Egger | 8 | -0.0097 | 0.0537 | 8.62E-01 |
| Transferrin | ieu-a-1052 | WM | 8 | -0.0354 | 0.0177 | 4.56E-02 |
| Triglycerides | ukb-d-30870_irnt | IVW | 192 | 0.2100 | 0.0560 | 1.75E-04 |
| Triglycerides | ukb-d-30870_irnt | MR Egger | 192 | -0.1072 | 0.0750 | 1.55E-01 |
| Triglycerides | ukb-d-30870_irnt | WM | 192 | 0.0922 | 0.0390 | 1.81E-02 |
| Trunk fat mass | ukb-a-291 | IVW | 265 | 0.5066 | 0.0700 | 4.67E-13 |
| Trunk fat mass | ukb-a-291 | MR Egger | 265 | 0.4583 | 0.2158 | 3.46E-02 |
| Trunk fat mass | ukb-a-291 | WM | 265 | 0.5763 | 0.0483 | 7.17E-33 |
| Trunk fat percentage | ukb-a-290 | IVW | 223 | 0.5532 | 0.0822 | 1.73E-11 |
| Trunk fat percentage | ukb-a-290 | MR Egger | 223 | 0.6232 | 0.2982 | 3.78E-02 |
| Trunk fat percentage | ukb-a-290 | WM | 223 | 0.5616 | 0.0615 | 6.48E-20 |

| | | | | | | |
|---|---|---|---|---|---|---|
| Trunk fat-free mass | ukb-a-292 | IVW | 384 | 0.0506 | 0.0691 | 4.64E-01 |
| Trunk fat-free mass | ukb-a-292 | MR Egger | 384 | -0.3869 | 0.1711 | 2.43E-02 |
| Trunk fat-free mass | ukb-a-292 | WM | 384 | -0.0590 | 0.0554 | 2.87E-01 |
| Trunk predicted mass | ukb-a-293 | IVW | 386 | 0.0502 | 0.0696 | 4.71E-01 |
| Trunk predicted mass | ukb-a-293 | MR Egger | 386 | -0.3923 | 0.1723 | 2.33E-02 |
| Trunk predicted mass | ukb-a-293 | WM | 386 | -0.0552 | 0.0574 | 3.36E-01 |
| Urate | ukb-d-30880_irnt | IVW | 205 | 0.1088 | 0.0552 | 4.88E-02 |
| Urate | ukb-d-30880_irnt | MR Egger | 205 | 0.0474 | 0.0766 | 5.36E-01 |
| Urate | ukb-d-30880_irnt | WM | 205 | 0.0611 | 0.0310 | 4.91E-02 |
| Urea | ukb-d-30670_irnt | IVW | 129 | 0.0170 | 0.0791 | 8.30E-01 |
| Urea | ukb-d-30670_irnt | MR Egger | 129 | -0.1833 | 0.1852 | 3.24E-01 |
| Urea | ukb-d-30670_irnt | WM | 129 | 0.0224 | 0.0570 | 6.95E-01 |
| Urinary sodium-potassium ratio | ieu-b-72 | IVW | 23 | -0.0363 | 0.2671 | 8.92E-01 |
| Urinary sodium-potassium ratio | ieu-b-72 | MR Egger | 23 | -2.8053 | 1.4762 | 7.12E-02 |
| Urinary sodium-potassium ratio | ieu-b-72 | WM | 23 | 0.1703 | 0.1829 | 3.52E-01 |
| Usual walking pace | ukb-a-513 | IVW | 28 | -1.6288 | 0.5024 | 1.19E-03 |
| Usual walking pace | ukb-a-513 | MR Egger | 28 | 0.0851 | 3.0549 | 9.78E-01 |
| Usual walking pace | ukb-a-513 | WM | 28 | -0.6055 | 0.2661 | 2.29E-02 |
| Vitamin D | ukb-d-30890_irnt | IVW | 55 | -0.0250 | 0.0575 | 6.64E-01 |
| Vitamin D | ukb-d-30890_irnt | MR Egger | 55 | 0.0166 | 0.0792 | 8.35E-01 |
| Vitamin D | ukb-d-30890_irnt | WM | 55 | -0.0218 | 0.0366 | 5.52E-01 |
| Waist circumference | ukb-a-382 | IVW | 214 | 0.9604 | 0.0785 | 2.17E-34 |
| Waist circumference | ukb-a-382 | MR Egger | 214 | 1.0869 | 0.2434 | 1.30E-05 |
| Waist circumference | ukb-a-382 | WM | 214 | 0.9400 | 0.0594 | 2.46E-56 |
| Waist-to-hip ratio | ieu-a-72 | IVW | 29 | 0.9746 | 0.2328 | 2.83E-05 |
| Waist-to-hip ratio | ieu-a-72 | MR Egger | 29 | 2.7659 | 1.0108 | 1.08E-02 |
| Waist-to-hip ratio | ieu-a-72 | WM | 29 | 0.7772 | 0.1354 | 9.40E-09 |
| Weight | ukb-a-249 | IVW | 319 | 0.4713 | 0.0661 | 1.00E-12 |
| Weight | ukb-a-249 | MR Egger | 319 | 0.2433 | 0.1749 | 1.65E-01 |
| Weight | ukb-a-249 | WM | 319 | 0.5499 | 0.0528 | 2.23E-25 |
| White blood cell (leukocyte) count | ukb-d-30000_irnt | IVW | 274 | -0.0346 | 0.0362 | 3.39E-01 |
| White blood cell (leukocyte) count | ukb-d-30000_irnt | MR Egger | 274 | -0.1219 | 0.0794 | 1.26E-01 |
| White blood cell (leukocyte) count | ukb-d-30000_irnt | WM | 274 | -0.0143 | 0.0401 | 7.22E-01 |
| Whole body fat mass | ukb-a-265 | IVW | 262 | 0.6149 | 0.0689 | 4.56E-19 |
| Whole body fat mass | ukb-a-265 | MR Egger | 262 | 0.7323 | 0.2099 | 5.68E-04 |
| Whole body fat mass | ukb-a-265 | WM | 262 | 0.6815 | 0.0488 | 2.27E-44 |
| Whole body fat-free mass | ukb-a-266 | IVW | 380 | 0.1712 | 0.0737 | 2.02E-02 |
| Whole body fat-free mass | ukb-a-266 | MR Egger | 380 | -0.3400 | 0.1796 | 5.91E-02 |
| Whole body fat-free mass | ukb-a-266 | WM | 380 | 0.0407 | 0.0606 | 5.02E-01 |
| Whole body water mass | ukb-a-267 | IVW | 376 | 0.1592 | 0.0731 | 2.93E-02 |
| Whole body water mass | ukb-a-267 | MR Egger | 376 | -0.3018 | 0.1768 | 8.88E-02 |
| Whole body water mass | ukb-a-267 | WM | 376 | 0.0382 | 0.0597 | 5.22E-01 |
