# Supplementary material for: Assessment of common risk factors of diabetes and chronic kidney disease: a Mendelian randomization study
Source: Front Endocrinol (Lausanne). 2023 Sep 13;14:1265719. doi: 10.3389/fendo.2023.1265719 (PMC10535100; doi:10.3389/fendo.2023.1265719)
Supplement: Supplementary file 3 [file Table_3.pdf]

Supplementary Table 3. MR analyses of the effect of exposures on CKD

Trait	Trait ID	Method	nsnp	Beta	SE	P
Adiponectin	ieu-a-1	IVW	14	0.0784	0.0693	2.58E-01
Adiponectin	ieu-a-1	MR Egger	14	0.0946	0.0999	3.62E-01
Adiponectin	ieu-a-1	WM	14	0.0745	0.0815	3.61E-01
Alanine aminotransferase	ukb-d-30620_irnt	IVW	166	0.0424	0.0715	5.53E-01
Alanine aminotransferase	ukb-d-30620_irnt	MR Egger	166	-0.0183	0.1328	8.91E-01
Alanine aminotransferase	ukb-d-30620_irnt	WM	166	-0.0767	0.1011	4.48E-01
Albumin	ukb-d-30600_irnt	IVW	171	0.1154	0.0691	9.50E-02
Albumin	ukb-d-30600_irnt	MR Egger	171	0.1402	0.1370	3.08E-01
Albumin	ukb-d-30600_irnt	WM	171	0.1123	0.0997	2.60E-01
Alcohol intake frequency	ukb-a-25	IVW	40	0.1394	0.1186	2.40E-01
Alcohol intake frequency	ukb-a-25	MR Egger	40	-0.2825	0.4558	5.39E-01
Alcohol intake frequency	ukb-a-25	WM	40	0.0949	0.1640	5.63E-01
Alcohol intake versus 10 years previously	ukb-a-32	IVW	7	-0.0388	0.5688	9.46E-01
Alcohol intake versus 10 years previously	ukb-a-32	MR Egger	7	-3.6855	6.5660	5.99E-01
Alcohol intake versus 10 years previously	ukb-a-32	WM	7	0.1832	0.7051	7.95E-01
Alcoholic drinks per week	ieu-b-73	IVW	32	-0.3515	0.2832	2.14E-01
Alcoholic drinks per week	ieu-b-73	MR Egger	32	-0.0933	0.8774	9.16E-01
Alcoholic drinks per week	ieu-b-73	WM	32	-0.0171	0.3382	9.60E-01
Alkaline phosphatase	ukb-d-30610_irnt	IVW	261	0.0072	0.0423	8.65E-01
Alkaline phosphatase	ukb-d-30610_irnt	MR Egger	261	-0.0379	0.0703	5.91E-01
Alkaline phosphatase	ukb-d-30610_irnt	WM	261	-0.0717	0.0687	2.96E-01
Apoliprotein A	ukb-d-30630_irnt	IVW	198	-0.1206	0.0509	1.78E-02
Apoliprotein A	ukb-d-30630_irnt	MR Egger	198	0.0747	0.0759	3.26E-01
Apoliprotein A	ukb-d-30630_irnt	WM	198	0.0000	0.0718	1.00E+00
Apoliprotein B	ukb-d-30640_irnt	IVW	132	-0.0507	0.0499	3.10E-01
Apoliprotein B	ukb-d-30640_irnt	MR Egger	132	-0.0818	0.0725	2.61E-01
Apoliprotein B	ukb-d-30640_irnt	WM	132	-0.1386	0.0692	4.51E-02
Aspartate aminotransferase	ukb-d-30650_irnt	IVW	197	-0.0328	0.0610	5.91E-01
Aspartate aminotransferase	ukb-d-30650_irnt	MR Egger	197	-0.1338	0.1140	2.42E-01
Aspartate aminotransferase	ukb-d-30650_irnt	WM	197	-0.1003	0.1009	3.20E-01
Basal metabolic rate	ukb-a-268	IVW	355	0.0945	0.0712	1.84E-01
Basal metabolic rate	ukb-a-268	MR Egger	355	-0.0505	0.1771	7.76E-01
Basal metabolic rate	ukb-a-268	WM	355	0.1433	0.1037	1.67E-01
Basophill percentage	ukb-d-30220_irnt	IVW	93	0.1092	0.0703	1.20E-01
Basophill percentage	ukb-d-30220_irnt	MR Egger	93	0.0708	0.1322	5.93E-01
Basophill percentage	ukb-d-30220_irnt	WM	93	0.0543	0.1154	6.38E-01
Birth weight	ukb-a-198	IVW	75	-0.1803	0.1000	7.15E-02
Birth weight	ukb-a-198	MR Egger	75	-0.5116	0.3505	1.49E-01
Birth weight	ukb-a-198	WM	75	-0.2105	0.1327	1.13E-01
Birth weight of first child	ukb-a-318	IVW	41	-0.0972	0.1107	3.80E-01
Birth weight of first child	ukb-a-318	MR Egger	41	0.6908	0.3893	8.38E-02
Birth weight of first child	ukb-a-318	WM	41	0.0275	0.1281	8.30E-01

Body fat	ieu-a-999	IVW	10	0.5714	0.2908	4.94E-02
Body fat	ieu-a-999	MR Egger	10	1.6036	1.3576	2.71E-01
Body fat	ieu-a-999	WM	10	0.7010	0.2591	6.82E-03
Body fat percentage	ukb-a-264	IVW	238	0.3674	0.0948	1.07E-04
Body fat percentage	ukb-a-264	MR Egger	238	0.6194	0.3486	7.69E-02
Body fat percentage	ukb-a-264	WM	238	0.3372	0.1219	5.67E-03
body mass index	ieu-b-40	IVW	476	0.4075	0.0574	1.29E-12
body mass index	ieu-b-40	MR Egger	476	0.4468	0.1520	3.45E-03
body mass index	ieu-b-40	WM	476	0.3897	0.0951	4.19E-05
Calcium	ukb-d-30680_irnt	IVW	172	-0.0111	0.0586	8.49E-01
Calcium	ukb-d-30680_irnt	MR Egger	172	-0.0124	0.1085	9.09E-01
Calcium	ukb-d-30680_irnt	WM	172	0.0113	0.1069	9.16E-01
Cholesterol	ukb-d-30690_irnt	IVW	139	-0.1255	0.0599	3.62E-02
Cholesterol	ukb-d-30690_irnt	MR Egger	139	-0.1028	0.0984	2.98E-01
Cholesterol	ukb-d-30690_irnt	WM	139	-0.1470	0.0796	6.50E-02
Cigarettes per Day	ieu-b-25	IVW	22	0.1659	0.0679	1.46E-02
Cigarettes per Day	ieu-b-25	MR Egger	22	0.0029	0.1134	9.80E-01
Cigarettes per Day	ieu-b-25	WM	22	0.1809	0.0865	3.64E-02
C-reactive protein	ukb-d-30710_irnt	IVW	170	0.1129	0.0458	1.36E-02
C-reactive protein	ukb-d-30710_irnt	MR Egger	170	0.0558	0.0632	3.79E-01
C-reactive protein	ukb-d-30710_irnt	WM	170	0.1731	0.0783	2.71E-02
Creatinine	ukb-d-30700_irnt	IVW	268	0.5455	0.0752	3.97E-13
Creatinine	ukb-d-30700_irnt	MR Egger	268	0.5495	0.1744	1.81E-03
Creatinine	ukb-d-30700_irnt	WM	268	0.4030	0.0929	1.45E-05
Creatinine (enzymatic) in urine	ukb-a-333	IVW	21	0.5946	0.4899	2.25E-01
Creatinine (enzymatic) in urine	ukb-a-333	MR Egger	21	-1.6159	2.0233	4.34E-01
Creatinine (enzymatic) in urine	ukb-a-333	WM	21	0.3809	0.3197	2.34E-01
Current tobacco smoking	ukb-a-16	IVW	16	0.8584	0.4915	8.07E-02
Current tobacco smoking	ukb-a-16	MR Egger	16	-1.1115	2.3048	6.37E-01
Current tobacco smoking	ukb-a-16	WM	16	1.1119	0.6766	1.00E-01
Cystatin C	ukb-d-30720_irnt	IVW	261	0.2524	0.0496	3.53E-07
Cystatin C	ukb-d-30720_irnt	MR Egger	261	0.1176	0.0675	8.25E-02
Cystatin C	ukb-d-30720_irnt	WM	261	0.0712	0.0513	1.65E-01
Daytime dozing / sleeping (narcolepsy)	ukb-a-15	IVW	19	-0.1171	0.5454	8.30E-01
Daytime dozing / sleeping (narcolepsy)	ukb-a-15	MR Egger	19	5.3176	2.5012	4.85E-02
Daytime dozing / sleeping (narcolepsy)	ukb-a-15	WM	19	0.0108	0.7217	9.88E-01
Diastolic blood pressure	ukb-a-359	IVW	165	0.1966	0.1093	7.20E-02
Diastolic blood pressure	ukb-a-359	MR Egger	165	0.1404	0.3833	7.15E-01
Diastolic blood pressure	ukb-a-359	WM	165	0.1163	0.1185	3.26E-01
Direct bilirubin	ukb-d-30660_irnt	IVW	63	0.0230	0.0288	4.24E-01
Direct bilirubin	ukb-d-30660_irnt	MR Egger	63	0.0316	0.0317	3.23E-01
Direct bilirubin	ukb-d-30660_irnt	WM	63	0.0207	0.0251	4.10E-01
Drive faster than motorway speed limit	ukb-a-8	IVW	13	-0.1439	0.3675	6.95E-01
Drive faster than motorway speed limit	ukb-a-8	MR Egger	13	0.9198	1.9528	6.47E-01
Drive faster than motorway speed limit	ukb-a-8	WM	13	0.0051	0.4665	9.91E-01

Eosinophill percentage	ukb-d-30210_irnt	IVW	266	0.1052	0.0420	1.23E-02
Eosinophill percentage	ukb-d-30210_irnt	MR Egger	266	0.1888	0.0862	2.94E-02
Eosinophill percentage	ukb-d-30210_irnt	WM	266	0.0802	0.0670	2.32E-01
Fasting glucose	ieu-b-114	IVW	30	0.3338	0.2080	1.08E-01
Fasting glucose	ieu-b-114	MR Egger	30	0.2391	0.4523	6.01E-01
Fasting glucose	ieu-b-114	WM	30	0.3928	0.2027	5.26E-02
Fasting insulin	ieu-b-116	IVW	14	0.4475	0.3081	1.46E-01
Fasting insulin	ieu-b-116	MR Egger	14	0.8705	1.6587	6.09E-01
Fasting insulin	ieu-b-116	WM	14	0.1511	0.4143	7.15E-01
Fluid intelligence score	ukb-a-196	IVW	42	-0.1372	0.0610	2.46E-02
Fluid intelligence score	ukb-a-196	MR Egger	42	0.3995	0.2714	1.49E-01
Fluid intelligence score	ukb-a-196	WM	42	-0.1330	0.0676	4.91E-02
Forced expiratory volume in 1-second (FEV1)	ukb-a-337	IVW	144	-0.1328	0.0993	1.81E-01
Forced expiratory volume in 1-second (FEV1)	ukb-a-337	MR Egger	144	-0.4295	0.3298	1.95E-01
Forced expiratory volume in 1-second (FEV1)	ukb-a-337	WM	144	-0.0316	0.1400	8.21E-01
Forced vital capacity (FVC)	ukb-a-336	IVW	202	-0.0899	0.0897	3.16E-01
Forced vital capacity (FVC)	ukb-a-336	MR Egger	202	-0.4027	0.2601	1.23E-01
Forced vital capacity (FVC)	ukb-a-336	WM	202	0.0422	0.1265	7.39E-01
Gamma glutamyltransferase	ukb-d-30730_irnt	IVW	227	-0.0226	0.0409	5.80E-01
Gamma glutamyltransferase	ukb-d-30730_irnt	MR Egger	227	0.0122	0.0656	8.53E-01
Gamma glutamyltransferase	ukb-d-30730_irnt	WM	227	0.1061	0.0638	9.64E-02
Getting up in morning	ukb-a-10	IVW	36	0.0993	0.2920	7.34E-01
Getting up in morning	ukb-a-10	MR Egger	36	1.1418	1.0414	2.81E-01
Getting up in morning	ukb-a-10	WM	36	0.1417	0.3520	6.87E-01
Glucose	ukb-d-30740_irnt	IVW	86	0.2246	0.0877	1.05E-02
Glucose	ukb-d-30740_irnt	MR Egger	86	0.2023	0.1435	1.62E-01
Glucose	ukb-d-30740_irnt	WM	86	0.1939	0.1052	6.53E-02
Glycated haemoglobin	ukb-d-30750_irnt	IVW	249	0.1519	0.0461	9.80E-04
Glycated haemoglobin	ukb-d-30750_irnt	MR Egger	249	0.0081	0.0769	9.16E-01
Glycated haemoglobin	ukb-d-30750_irnt	WM	249	0.0663	0.0675	3.26E-01
Haematocrit percentage	ukb-d-30030_irnt	IVW	211	-0.1654	0.0718	2.12E-02
Haematocrit percentage	ukb-d-30030_irnt	MR Egger	211	0.1156	0.1513	4.46E-01
Haematocrit percentage	ukb-d-30030_irnt	WM	211	-0.0300	0.1094	7.84E-01
Haemoglobin concentration	ukb-d-30020_irnt	IVW	232	-0.1080	0.0681	1.13E-01
Haemoglobin concentration	ukb-d-30020_irnt	MR Egger	232	0.1302	0.1386	3.49E-01
Haemoglobin concentration	ukb-d-30020_irnt	WM	232	-0.0329	0.1129	7.71E-01
HDL cholesterol	ukb-d-30760_irnt	IVW	222	-0.1512	0.0437	5.42E-04
HDL cholesterol	ukb-d-30760_irnt	MR Egger	222	0.0035	0.0630	9.56E-01
HDL cholesterol	ukb-d-30760_irnt	WM	222	-0.0011	0.0650	9.86E-01
Heart rate	ieu-a-1056	IVW	14	0.0072	0.0131	5.82E-01
Heart rate	ieu-a-1056	MR Egger	14	0.0835	0.0486	1.11E-01
Heart rate	ieu-a-1056	WM	14	0.0064	0.0173	7.13E-01
Heel bone mineral density (BMD) T-score	ukb-a-500	IVW	232	0.0008	0.0380	9.82E-01
Heel bone mineral density (BMD) T-score	ukb-a-500	MR Egger	232	-0.0487	0.0733	5.07E-01
Heel bone mineral density (BMD) T-score	ukb-a-500	WM	232	-0.0692	0.0616	2.61E-01

High light scatter reticulocyte count	ukb-d-30300_irnt	IVW	250	0.0902	0.0455	4.77E-02
High light scatter reticulocyte count	ukb-d-30300_irnt	MR Egger	250	0.0586	0.0887	5.10E-01
High light scatter reticulocyte count	ukb-d-30300_irnt	WM	250	0.0543	0.0741	4.63E-01
High light scatter reticulocyte percentage	ukb-d-30290_irnt	IVW	260	0.0955	0.0452	3.46E-02
High light scatter reticulocyte percentage	ukb-d-30290_irnt	MR Egger	260	0.0561	0.0861	5.15E-01
High light scatter reticulocyte percentage	ukb-d-30290_irnt	WM	260	0.0394	0.0678	5.61E-01
Hip circumference	ukb-a-388	IVW	270	0.2087	0.0599	4.95E-04
Hip circumference	ukb-a-388	MR Egger	270	0.3989	0.1711	2.05E-02
Hip circumference	ukb-a-388	WM	270	0.2015	0.0859	1.89E-02
IGF-1	ukb-d-30770_irnt	IVW	288	-0.0076	0.0431	8.61E-01
IGF-1	ukb-d-30770_irnt	MR Egger	288	0.0490	0.0843	5.61E-01
IGF-1	ukb-d-30770_irnt	WM	288	-0.0136	0.0669	8.39E-01
Immature reticulocyte fraction	ukb-d-30280_irnt	IVW	181	0.0630	0.0535	2.39E-01
Immature reticulocyte fraction	ukb-d-30280_irnt	MR Egger	181	-0.1160	0.0965	2.31E-01
Immature reticulocyte fraction	ukb-d-30280_irnt	WM	181	-0.0027	0.0811	9.73E-01
Impedance of whole body	ukb-a-269	IVW	333	-0.1362	0.0708	5.44E-02
Impedance of whole body	ukb-a-269	MR Egger	333	-0.1008	0.1989	6.13E-01
Impedance of whole body	ukb-a-269	WM	333	-0.0521	0.1061	6.23E-01
Job involves mainly walking or standing	ukb-a-502	IVW	7	0.2264	0.2723	4.06E-01
Job involves mainly walking or standing	ukb-a-502	MR Egger	7	0.6303	2.7401	8.27E-01
Job involves mainly walking or standing	ukb-a-502	WM	7	0.2132	0.3280	5.16E-01
LDL direct	ukb-d-30780_irnt	IVW	128	-0.0525	0.0550	3.40E-01
LDL direct	ukb-d-30780_irnt	MR Egger	128	-0.1040	0.0829	2.12E-01
LDL direct	ukb-d-30780_irnt	WM	128	-0.1343	0.0815	9.92E-02
Length of menstrual cycle	ukb-a-351	IVW	6	0.1465	0.1169	2.10E-01
Length of menstrual cycle	ukb-a-351	MR Egger	6	0.4663	0.2574	1.44E-01
Length of menstrual cycle	ukb-a-351	WM	6	0.2704	0.1431	5.88E-02
Lipoprotein A	ukb-d-30790_irnt	IVW	17	0.0445	0.0215	3.83E-02
Lipoprotein A	ukb-d-30790_irnt	MR Egger	17	0.0193	0.0264	4.77E-01
Lipoprotein A	ukb-d-30790_irnt	WM	17	0.0216	0.0255	3.96E-01
Lymphocyte count	ukb-d-30120_irnt	IVW	279	-0.0190	0.0492	7.00E-01
Lymphocyte count	ukb-d-30120_irnt	MR Egger	279	-0.0476	0.1174	6.86E-01
Lymphocyte count	ukb-d-30120_irnt	WM	279	0.0177	0.0773	8.19E-01
Lymphocyte percentage	ukb-d-30180_irnt	IVW	238	-0.0394	0.0640	5.38E-01
Lymphocyte percentage	ukb-d-30180_irnt	MR Egger	238	-0.1723	0.1422	2.27E-01
Lymphocyte percentage	ukb-d-30180_irnt	WM	238	-0.0833	0.0804	3.00E-01
Mean corpuscular haemoglobin	ukb-d-30050_irnt	IVW	295	-0.0217	0.0333	5.16E-01
Mean corpuscular haemoglobin	ukb-d-30050_irnt	MR Egger	295	0.0137	0.0540	8.01E-01
Mean corpuscular haemoglobin	ukb-d-30050_irnt	WM	295	-0.0314	0.0537	5.59E-01
Mean corpuscular haemoglobin concentration	ukb-d-30060_irnt	IVW	84	-0.0263	0.0875	7.64E-01
Mean corpuscular haemoglobin concentration	ukb-d-30060_irnt	MR Egger	84	0.1089	0.1725	5.29E-01
Mean corpuscular haemoglobin concentration	ukb-d-30060_irnt	WM	84	-0.0761	0.1167	5.14E-01
Mean corpuscular volume	ukb-d-30040_irnt	IVW	301	-0.0093	0.0325	7.74E-01
Mean corpuscular volume	ukb-d-30040_irnt	MR Egger	301	0.0069	0.0554	9.01E-01
Mean corpuscular volume	ukb-d-30040_irnt	WM	301	-0.0334	0.0543	5.38E-01

Mean platelet (thrombocyte) volume	ukb-d-30100_irnt	IVW	356	-0.0164	0.0241	4.96E-01
Mean platelet (thrombocyte) volume	ukb-d-30100_irnt	MR Egger	356	-0.0323	0.0360	3.70E-01
Mean platelet (thrombocyte) volume	ukb-d-30100_irnt	WM	356	-0.0149	0.0422	7.23E-01
Mean reticulocyte volume	ukb-d-30260_irnt	IVW	268	-0.0126	0.0397	7.50E-01
Mean reticulocyte volume	ukb-d-30260_irnt	MR Egger	268	-0.0652	0.0717	3.64E-01
Mean reticulocyte volume	ukb-d-30260_irnt	WM	268	-0.0603	0.0561	2.83E-01
Mean sphered cell volume	ukb-d-30270_irnt	IVW	276	-0.0155	0.0388	6.89E-01
Mean sphered cell volume	ukb-d-30270_irnt	MR Egger	276	-0.0471	0.0684	4.92E-01
Mean sphered cell volume	ukb-d-30270_irnt	WM	276	0.0188	0.0580	7.46E-01
Mean time to correctly identify matches	ukb-a-199	IVW	26	-0.5483	0.2843	5.37E-02
Mean time to correctly identify matches	ukb-a-199	MR Egger	26	-3.4640	1.7984	6.60E-02
Mean time to correctly identify matches	ukb-a-199	WM	26	-0.7061	0.3116	2.34E-02
Microalbumin in urine	ukb-d-30500_irnt	IVW	4	-0.0290	0.2709	9.15E-01
Microalbumin in urine	ukb-d-30500_irnt	MR Egger	4	0.3851	0.5287	5.42E-01
Microalbumin in urine	ukb-d-30500_irnt	WM	4	-0.0713	0.3100	8.18E-01
Monocyte count	ukb-d-30130_irnt	IVW	277	-0.0596	0.0439	1.75E-01
Monocyte count	ukb-d-30130_irnt	MR Egger	277	-0.0229	0.0714	7.49E-01
Monocyte count	ukb-d-30130_irnt	WM	277	-0.0303	0.0583	6.03E-01
Monocyte percentage	ukb-d-30190_irnt	IVW	257	0.0036	0.0373	9.23E-01
Monocyte percentage	ukb-d-30190_irnt	MR Egger	257	-0.0366	0.0623	5.58E-01
Monocyte percentage	ukb-d-30190_irnt	WM	257	-0.0026	0.0609	9.66E-01
Morning/evening person (chronotype)	ukb-a-11	IVW	80	-0.3289	0.1267	9.41E-03
Morning/evening person (chronotype)	ukb-a-11	MR Egger	80	-0.5028	0.2952	9.25E-02
Morning/evening person (chronotype)	ukb-a-11	WM	80	-0.3237	0.1707	5.79E-02
Nap during day	ukb-a-12	IVW	47	0.4000	0.2599	1.24E-01
Nap during day	ukb-a-12	MR Egger	47	1.1228	0.9858	2.61E-01
Nap during day	ukb-a-12	WM	47	0.6261	0.3646	8.60E-02
Neuroticism	ieu-a-1007	IVW	9	0.2795	0.2991	3.50E-01
Neuroticism	ieu-a-1007	MR Egger	9	-4.5776	2.1660	7.24E-02
Neuroticism	ieu-a-1007	WM	9	0.4675	0.3653	2.01E-01
Neuroticism score	ukb-a-230	IVW	62	0.0177	0.0455	6.98E-01
Neuroticism score	ukb-a-230	MR Egger	62	-0.2899	0.2657	2.80E-01
Neuroticism score	ukb-a-230	WM	62	0.0040	0.0589	9.46E-01
Neutrophill count	ukb-d-30140_irnt	IVW	239	0.0066	0.0527	9.01E-01
Neutrophill count	ukb-d-30140_irnt	MR Egger	239	-0.1025	0.1116	3.59E-01
Neutrophill count	ukb-d-30140_irnt	WM	239	0.0298	0.0810	7.13E-01
Neutrophill percentage	ukb-d-30200_irnt	IVW	238	-0.0616	0.0523	2.39E-01
Neutrophill percentage	ukb-d-30200_irnt	MR Egger	238	0.0862	0.1179	4.65E-01
Neutrophill percentage	ukb-d-30200_irnt	WM	238	0.0350	0.0794	6.59E-01
Overall health rating	ukb-a-251	IVW	49	0.2457	0.2374	3.01E-01
Overall health rating	ukb-a-251	MR Egger	49	0.6886	1.4134	6.28E-01
Overall health rating	ukb-a-251	WM	49	0.4349	0.3112	1.62E-01
Past tobacco smoking	ukb-a-17	IVW	40	0.0767	0.1644	6.41E-01
Past tobacco smoking	ukb-a-17	MR Egger	40	0.6572	0.6597	3.25E-01
Past tobacco smoking	ukb-a-17	WM	40	0.1434	0.1953	4.63E-01

Peak expiratory flow (PEF)	ukb-a-338	IVW	78	-0.1289	0.1409	3.60E-01
Peak expiratory flow (PEF)	ukb-a-338	MR Egger	78	0.1347	0.5151	7.94E-01
Peak expiratory flow (PEF)	ukb-a-338	WM	78	0.0425	0.1956	8.28E-01
Phosphate	ukb-d-30810_irnt	IVW	134	0.0794	0.0556	1.53E-01
Phosphate	ukb-d-30810_irnt	MR Egger	134	0.0077	0.0916	9.33E-01
Phosphate	ukb-d-30810_irnt	WM	134	0.0086	0.0923	9.26E-01
Platelet count	ukb-d-30080_irnt	IVW	350	0.0110	0.0344	7.50E-01
Platelet count	ukb-d-30080_irnt	MR Egger	350	0.0497	0.0615	4.20E-01
Platelet count	ukb-d-30080_irnt	WM	350	0.0236	0.0567	6.78E-01
Platelet crit	ukb-d-30090_irnt	IVW	317	0.0028	0.0404	9.45E-01
Platelet crit	ukb-d-30090_irnt	MR Egger	317	0.1913	0.0712	7.63E-03
Platelet crit	ukb-d-30090_irnt	WM	317	0.0243	0.0596	6.83E-01
Platelet distribution width	ukb-d-30110_irnt	IVW	283	0.0307	0.0301	3.07E-01
Platelet distribution width	ukb-d-30110_irnt	MR Egger	283	0.0066	0.0442	8.81E-01
Platelet distribution width	ukb-d-30110_irnt	WM	283	-0.0452	0.0495	3.62E-01
Potassium in urine	ukb-a-334	IVW	9	0.0227	0.6215	9.71E-01
Potassium in urine	ukb-a-334	MR Egger	9	-6.8972	3.7866	1.11E-01
Potassium in urine	ukb-a-334	WM	9	0.5428	0.5320	3.08E-01
Pulse rate	ukb-a-3	IVW	189	0.1073	0.0621	8.42E-02
Pulse rate	ukb-a-3	MR Egger	189	-0.0710	0.1462	6.28E-01
Pulse rate	ukb-a-3	WM	189	0.0586	0.0860	4.95E-01
QRS duration	ukb-d-12340_irnt	IVW	5	-0.1001	0.0823	2.23E-01
QRS duration	ukb-d-12340_irnt	MR Egger	5	0.0518	0.3633	8.96E-01
QRS duration	ukb-d-12340_irnt	WM	5	-0.0911	0.1046	3.84E-01
Red blood cell (erythrocyte) count	ukb-d-30010_irnt	IVW	293	-0.0169	0.0533	7.51E-01
Red blood cell (erythrocyte) count	ukb-d-30010_irnt	MR Egger	293	-0.0729	0.1002	4.68E-01
Red blood cell (erythrocyte) count	ukb-d-30010_irnt	WM	293	0.0331	0.0936	7.24E-01
Red blood cell (erythrocyte) distribution width	ukb-d-30070_irnt	IVW	246	0.0373	0.0411	3.65E-01
Red blood cell (erythrocyte) distribution width	ukb-d-30070_irnt	MR Egger	246	0.0373	0.0734	6.11E-01
Red blood cell (erythrocyte) distribution width	ukb-d-30070_irnt	WM	246	0.0455	0.0637	4.75E-01
Reticulocyte count	ukb-d-30250_irnt	IVW	237	0.0943	0.0468	4.38E-02
Reticulocyte count	ukb-d-30250_irnt	MR Egger	237	0.0711	0.0863	4.11E-01
Reticulocyte count	ukb-d-30250_irnt	WM	237	0.1007	0.0723	1.64E-01
Reticulocyte percentage	ukb-d-30240_irnt	IVW	227	0.0901	0.0472	5.65E-02
Reticulocyte percentage	ukb-d-30240_irnt	MR Egger	227	0.0248	0.0882	7.79E-01
Reticulocyte percentage	ukb-d-30240_irnt	WM	227	0.0980	0.0693	1.57E-01
Serum cystatin C (eGFRcys)	ieu-a-1106	IVW	5	-0.9571	1.0265	3.51E-01
Serum cystatin C (eGFRcys)	ieu-a-1106	MR Egger	5	0.0383	1.5857	9.82E-01
Serum cystatin C (eGFRcys)	ieu-a-1106	WM	5	-0.4555	0.2770	1.00E-01
SHBG	ukb-d-30830_irnt	IVW	221	-0.1113	0.0394	4.69E-03
SHBG	ukb-d-30830_irnt	MR Egger	221	-0.0329	0.0598	5.83E-01
SHBG	ukb-d-30830_irnt	WM	221	-0.0631	0.0678	3.52E-01
Sitting height	ukb-a-195	IVW	411	-0.0719	0.0474	1.29E-01
Sitting height	ukb-a-195	MR Egger	411	-0.0077	0.1180	9.48E-01
Sitting height	ukb-a-195	WM	411	0.0038	0.0752	9.60E-01

Sleep duration	ukb-a-9	IVW	41	-0.0466	0.2306	8.40E-01
Sleep duration	ukb-a-9	MR Egger	41	-1.1223	0.9259	2.33E-01
Sleep duration	ukb-a-9	WM	41	-0.1044	0.3272	7.50E-01
Sleeplessness / insomnia	ukb-a-13	IVW	28	-0.0855	0.3094	7.82E-01
Sleeplessness / insomnia	ukb-a-13	MR Egger	28	0.5451	0.8932	5.47E-01
Sleeplessness / insomnia	ukb-a-13	WM	28	-0.0988	0.4220	8.15E-01
Sodium in urine	ukb-a-335	IVW	29	0.1467	0.2009	4.65E-01
Sodium in urine	ukb-a-335	MR Egger	29	1.2333	0.9115	1.87E-01
Sodium in urine	ukb-a-335	WM	29	0.0129	0.2776	9.63E-01
Standing height	ukb-a-389	IVW	564	-0.1085	0.0402	6.98E-03
Standing height	ukb-a-389	MR Egger	564	-0.0717	0.0879	4.15E-01
Standing height	ukb-a-389	WM	564	-0.0565	0.0618	3.61E-01
Systolic blood pressure	ukb-a-360	IVW	145	0.2835	0.0886	1.38E-03
Systolic blood pressure	ukb-a-360	MR Egger	145	0.4569	0.2957	1.25E-01
Systolic blood pressure	ukb-a-360	WM	145	0.1975	0.1196	9.88E-02
telomere length	ieu-b-4879	IVW	129	-0.0790	0.0796	3.21E-01
telomere length	ieu-b-4879	MR Egger	129	-0.1015	0.1410	4.73E-01
telomere length	ieu-b-4879	WM	129	0.0113	0.1057	9.15E-01
Testosterone	ukb-d-30850_irnt	IVW	83	-0.2716	0.1624	9.45E-02
Testosterone	ukb-d-30850_irnt	MR Egger	83	-0.0525	0.2804	8.52E-01
Testosterone	ukb-d-30850_irnt	WM	83	-0.1811	0.2335	4.38E-01
Total bilirubin	ukb-d-30840_irnt	IVW	113	0.0158	0.0271	5.61E-01
Total bilirubin	ukb-d-30840_irnt	MR Egger	113	0.0271	0.0291	3.54E-01
Total bilirubin	ukb-d-30840_irnt	WM	113	0.0150	0.0225	5.05E-01
Total cholesterol	ieu-a-301	IVW	83	-0.1629	0.0452	3.17E-04
Total cholesterol	ieu-a-301	MR Egger	83	-0.1590	0.0734	3.32E-02
Total cholesterol	ieu-a-301	WM	83	-0.1612	0.0655	1.38E-02
Total protein	ukb-d-30860_irnt	IVW	193	0.1432	0.0612	1.94E-02
Total protein	ukb-d-30860_irnt	MR Egger	193	-0.0668	0.1324	6.14E-01
Total protein	ukb-d-30860_irnt	WM	193	0.1009	0.0933	2.79E-01
Townsend deprivation index at recruitment	ukb-a-44	IVW	5	0.2121	0.5181	6.82E-01
Townsend deprivation index at recruitment	ukb-a-44	MR Egger	5	-3.7057	2.4126	2.22E-01
Townsend deprivation index at recruitment	ukb-a-44	WM	5	0.6935	0.6960	3.19E-01
Transferrin	ieu-a-1052	IVW	8	0.0234	0.0697	7.37E-01
Transferrin	ieu-a-1052	MR Egger	8	-0.0282	0.1088	8.04E-01
Transferrin	ieu-a-1052	WM	8	0.0379	0.0413	3.59E-01
Triglycerides	ukb-d-30870_irnt	IVW	192	0.1487	0.0430	5.47E-04
Triglycerides	ukb-d-30870_irnt	MR Egger	192	0.0880	0.0622	1.59E-01
Triglycerides	ukb-d-30870_irnt	WM	192	0.2040	0.0649	1.66E-03
Trunk fat mass	ukb-a-291	IVW	265	0.2591	0.0642	5.50E-05
Trunk fat mass	ukb-a-291	MR Egger	265	0.3428	0.1976	8.39E-02
Trunk fat mass	ukb-a-291	WM	265	0.2404	0.0835	3.97E-03
Trunk fat percentage	ukb-a-290	IVW	223	0.2176	0.0853	1.08E-02
Trunk fat percentage	ukb-a-290	MR Egger	223	0.1576	0.3095	6.11E-01
Trunk fat percentage	ukb-a-290	WM	223	0.0984	0.1059	3.53E-01

Trunk fat-free mass	ukb-a-292	IVW	384	0.0094	0.0694	8.92E-01
Trunk fat-free mass	ukb-a-292	MR Egger	384	0.0230	0.1732	8.94E-01
Trunk fat-free mass	ukb-a-292	WM	384	0.0633	0.0991	5.23E-01
Trunk predicted mass	ukb-a-293	IVW	386	0.0122	0.0694	8.60E-01
Trunk predicted mass	ukb-a-293	MR Egger	386	0.0488	0.1730	7.78E-01
Trunk predicted mass	ukb-a-293	WM	386	0.0649	0.1001	5.17E-01
Urate	ukb-d-30880_irnt	IVW	205	0.2510	0.0519	1.32E-06
Urate	ukb-d-30880_irnt	MR Egger	205	0.1531	0.0716	3.38E-02
Urate	ukb-d-30880_irnt	WM	205	0.0983	0.0707	1.64E-01
Urea	ukb-d-30670_irnt	IVW	129	0.3083	0.1078	4.23E-03
Urea	ukb-d-30670_irnt	MR Egger	129	0.2040	0.2539	4.23E-01
Urea	ukb-d-30670_irnt	WM	129	0.1482	0.1251	2.36E-01
Urinary sodium-potassium ratio	ieu-b-72	IVW	23	0.1447	0.2936	6.22E-01
Urinary sodium-potassium ratio	ieu-b-72	MR Egger	23	-0.7693	1.7449	6.64E-01
Urinary sodium-potassium ratio	ieu-b-72	WM	23	-0.0322	0.3174	9.19E-01
Usual walking pace	ukb-a-513	IVW	28	-1.1523	0.3843	2.71E-03
Usual walking pace	ukb-a-513	MR Egger	28	0.6906	2.3145	7.68E-01
Usual walking pace	ukb-a-513	WM	28	-1.2661	0.5152	1.40E-02
Vitamin D	ukb-d-30890_irnt	IVW	55	-0.0506	0.0603	4.01E-01
Vitamin D	ukb-d-30890_irnt	MR Egger	55	0.0014	0.0826	9.87E-01
Vitamin D	ukb-d-30890_irnt	WM	55	-0.0635	0.0771	4.10E-01
Waist circumference	ukb-a-382	IVW	214	0.3467	0.0789	1.10E-05
Waist circumference	ukb-a-382	MR Egger	214	0.5846	0.2439	1.74E-02
Waist circumference	ukb-a-382	WM	214	0.2832	0.1111	1.08E-02
Waist-to-hip ratio	ieu-a-72	IVW	29	0.3379	0.1897	7.49E-02
Waist-to-hip ratio	ieu-a-72	MR Egger	29	0.5796	0.8726	5.12E-01
Waist-to-hip ratio	ieu-a-72	WM	29	0.1997	0.2136	3.50E-01
Weight	ukb-a-249	IVW	319	0.2158	0.0573	1.65E-04
Weight	ukb-a-249	MR Egger	319	0.1953	0.1516	1.99E-01
Weight	ukb-a-249	WM	319	0.2100	0.0868	1.56E-02
White blood cell (leukocyte) count	ukb-d-30000_irnt	IVW	274	0.0020	0.0520	9.69E-01
White blood cell (leukocyte) count	ukb-d-30000_irnt	MR Egger	274	-0.1011	0.1142	3.77E-01
White blood cell (leukocyte) count	ukb-d-30000_irnt	WM	274	0.0252	0.0758	7.39E-01
Whole body fat mass	ukb-a-265	IVW	262	0.3414	0.0620	3.74E-08
Whole body fat mass	ukb-a-265	MR Egger	262	0.3872	0.1889	4.13E-02
Whole body fat mass	ukb-a-265	WM	262	0.2929	0.0923	1.50E-03
Whole body fat-free mass	ukb-a-266	IVW	380	-0.0139	0.0706	8.44E-01
Whole body fat-free mass	ukb-a-266	MR Egger	380	-0.0379	0.1737	8.28E-01
Whole body fat-free mass	ukb-a-266	WM	380	0.0627	0.1034	5.45E-01
Whole body water mass	ukb-a-267	IVW	376	0.0224	0.0708	7.52E-01
Whole body water mass	ukb-a-267	MR Egger	376	-0.0212	0.1728	9.02E-01
Whole body water mass	ukb-a-267	WM	376	0.0945	0.1030	3.59E-01
