# Supplementary material for: Assessment of common risk factors of diabetes and chronic kidney disease: a Mendelian randomization study
Source: Front Endocrinol (Lausanne). 2023 Sep 13;14:1265719. doi: 10.3389/fendo.2023.1265719 (PMC10535100; doi:10.3389/fendo.2023.1265719)
Supplement: Supplementary file 4 [file Table_4.pdf]

Supplementary Table 4. The results of horizontal pleiotropy testing

Trait	Trait ID	Disease	Egger_intercept	SE	P
Adiponectin	ieu-a-1	T1D	0.0183	0.0116	1.39E-01
Alanine aminotransferase	ukb-d-30620_irnt	T1D	0.0008	0.0084	9.22E-01
Albumin	ukb-d-30600_irnt	T1D	0.0020	0.0063	7.51E-01
Alcohol intake frequency	ukb-a-25	T1D	-0.0112	0.0159	4.87E-01
Alcohol intake versus 10 years previously	ukb-a-32	T1D	0.0121	0.1308	9.30E-01
Alcoholic drinks per week	ieu-b-73	T1D	0.0149	0.0261	5.71E-01
Alkaline phosphatase	ukb-d-30610_irnt	T1D	0.0105	0.0066	1.15E-01
Apoliprotein A	ukb-d-30630_irnt	T1D	-0.0066	0.0065	3.10E-01
Apoliprotein B	ukb-d-30640_irnt	T1D	0.0031	0.0047	5.12E-01
Aspartate aminotransferase	ukb-d-30650_irnt	T1D	-0.0034	0.0063	5.86E-01
Basal metabolic rate	ukb-a-268	T1D	-0.0048	0.0058	4.16E-01
Basophill percentage	ukb-d-30220_irnt	T1D	0.0086	0.0119	4.68E-01
Birth weight	ukb-a-198	T1D	-0.0011	0.0132	9.36E-01
Birth weight of first child	ukb-a-318	T1D	0.0200	0.0320	5.34E-01
Body fat	ieu-a-999	T1D	-0.0109	0.0667	8.74E-01
Body fat percentage	ukb-a-264	T1D	-0.0029	0.0091	7.53E-01
body mass index	ieu-b-40	T1D	-0.0090	0.0039	2.28E-02
Calcium	ukb-d-30680_irnt	T1D	-0.0016	0.0070	8.26E-01
Cholesterol	ukb-d-30690_irnt	T1D	-0.0095	0.0109	3.84E-01
Cigarettes per Day	ieu-b-25	T1D	-0.0046	0.0103	6.59E-01
C-reactive protein	ukb-d-30710_irnt	T1D	-0.0007	0.0121	9.57E-01
Creatinine	ukb-d-30700_irnt	T1D	0.0003	0.0049	9.59E-01
Creatinine (enzymatic) in urine	ukb-a-333	T1D	-0.0230	0.0372	5.43E-01
Current tobacco smoking	ukb-a-16	T1D	-0.0409	0.0380	3.00E-01
Cystatin C	ukb-d-30720_irnt	T1D	0.0066	0.0032	4.11E-02
Daytime dozing / sleeping (narcolepsy)	ukb-a-15	T1D	-0.0363	0.0359	3.25E-01
Diastolic blood pressure	ukb-a-359	T1D	-0.0054	0.0100	5.88E-01
Direct bilirubin	ukb-d-30660_irnt	T1D	-0.0018	0.0054	7.39E-01
Drive faster than motorway speed limit	ukb-a-8	T1D	-0.2274	0.1611	1.86E-01
Eosinophill percentage	ukb-d-30210_irnt	T1D	0.0016	0.0060	7.97E-01
Fasting glucose	ieu-b-114	T1D	0.0265	0.0156	1.00E-01
Fasting insulin	ieu-b-116	T1D	0.0138	0.0401	7.36E-01
Fluid intelligence score	ukb-a-196	T1D	-0.0013	0.0383	9.73E-01
Forced expiratory volume in 1-second (FEV1)	ukb-a-337	T1D	0.0011	0.0143	9.40E-01
Forced vital capacity (FVC)	ukb-a-336	T1D	0.0281	0.0113	1.38E-02
Gamma glutamyltransferase	ukb-d-30730_irnt	T1D	-0.0016	0.0042	6.97E-01
Getting up in morning	ukb-a-10	T1D	-0.0208	0.0169	2.27E-01
Glucose	ukb-d-30740_irnt	T1D	0.0062	0.0173	7.22E-01
Glycated haemoglobin	ukb-d-30750_irnt	T1D	-0.0056	0.0088	5.22E-01
Haematocrit percentage	ukb-d-30030_irnt	T1D	-0.0011	0.0049	8.28E-01
Haemoglobin concentration	ukb-d-30020_irnt	T1D	-0.0002	0.0047	9.58E-01
HDL cholesterol	ukb-d-30760_irnt	T1D	0.0021	0.0060	7.28E-01

Heart rate	ieu-a-1056	T1D	0.0182	0.0294	5.47E-01
Heel bone mineral density (BMD) T-score	ukb-a-500	T1D	-0.0082	0.0041	4.68E-02
High light scatter reticulocyte count	ukb-d-30300_irnt	T1D	0.0097	0.0080	2.24E-01
High light scatter reticulocyte percentage	ukb-d-30290_irnt	T1D	0.0000	0.0052	9.97E-01
Hip circumference	ukb-a-388	T1D	-0.0141	0.0063	2.64E-02
IGF-1	ukb-d-30770_irnt	T1D	0.0024	0.0042	5.63E-01
Immature reticulocyte fraction	ukb-d-30280_irnt	T1D	-0.0097	0.0067	1.48E-01
Impedance of whole body	ukb-a-269	T1D	-0.0003	0.0055	9.52E-01
Job involves mainly walking or standing	ukb-a-502	T1D	-0.0456	0.1291	7.38E-01
LDL direct	ukb-d-30780_irnt	T1D	-0.0005	0.0057	9.30E-01
Length of menstrual cycle	ukb-a-351	T1D	0.0241	0.0237	3.67E-01
Lipoprotein A	ukb-d-30790_irnt	T1D	0.0168	0.0137	2.41E-01
Lymphocyte count	ukb-d-30120_irnt	T1D	0.0047	0.0075	5.28E-01
Lymphocyte percentage	ukb-d-30180_irnt	T1D	0.0100	0.0095	2.93E-01
Mean corpuscular haemoglobin	ukb-d-30050_irnt	T1D	0.0012	0.0033	7.13E-01
Mean corpuscular haemoglobin concentration	ukb-d-30060_irnt	T1D	0.0085	0.0116	4.67E-01
Mean corpuscular volume	ukb-d-30040_irnt	T1D	0.0052	0.0040	1.86E-01
Mean platelet (thrombocyte) volume	ukb-d-30100_irnt	T1D	-0.0009	0.0040	8.16E-01
Mean reticulocyte volume	ukb-d-30260_irnt	T1D	0.0003	0.0059	9.55E-01
Mean sphered cell volume	ukb-d-30270_irnt	T1D	-0.0050	0.0060	4.09E-01
Mean time to correctly identify matches	ukb-a-199	T1D	0.0429	0.0437	3.36E-01
Microalbumin in urine	ukb-d-30500_irnt	T1D	0.0405	0.0385	4.03E-01
Monocyte count	ukb-d-30130_irnt	T1D	0.0145	0.0083	8.11E-02
Monocyte percentage	ukb-d-30190_irnt	T1D	0.0088	0.0045	5.01E-02
Morning/evening person (chronotype)	ukb-a-11	T1D	0.0116	0.0091	2.03E-01
Nap during day	ukb-a-12	T1D	-0.0077	0.0147	6.05E-01
Neuroticism	ieu-a-1007	T1D	-0.0103	0.0873	9.09E-01
Neuroticism score	ukb-a-230	T1D	0.0027	0.0309	9.32E-01
Neutrophill count	ukb-d-30140_irnt	T1D	0.0008	0.0068	9.08E-01
Neutrophill percentage	ukb-d-30200_irnt	T1D	-0.0009	0.0079	9.05E-01
Overall health rating	ukb-a-251	T1D	0.0007	0.0286	9.80E-01
Past tobacco smoking	ukb-a-17	T1D	0.0019	0.0293	9.49E-01
Peak expiratory flow (PEF)	ukb-a-338	T1D	0.0346	0.0225	1.27E-01
Phosphate	ukb-d-30810_irnt	T1D	0.0037	0.0055	5.05E-01
Platelet count	ukb-d-30080_irnt	T1D	-0.0080	0.0034	1.83E-02
Platelet crit	ukb-d-30090_irnt	T1D	-0.0057	0.0045	2.07E-01
Platelet distribution width	ukb-d-30110_irnt	T1D	-0.0006	0.0030	8.32E-01
Potassium in urine	ukb-a-334	T1D	0.4359	0.1792	4.52E-02
Pulse rate	ukb-a-3	T1D	-0.0041	0.0060	4.95E-01
QRS duration	ukb-d-12340_irnt	T1D	-0.0595	0.0968	5.82E-01
Red blood cell (erythrocyte) count	ukb-d-30010_irnt	T1D	-0.0008	0.0047	8.64E-01
Red blood cell (erythrocyte) distribution width	ukb-d-30070_irnt	T1D	-0.0060	0.0040	1.32E-01
Reticulocyte count	ukb-d-30250_irnt	T1D	0.0024	0.0044	5.93E-01
Reticulocyte percentage	ukb-d-30240_irnt	T1D	0.0043	0.0063	5.01E-01
Serum cystatin C (eGFRcys)	ieu-a-1106	T1D	-0.0339	0.0540	5.75E-01

SHBG	ukb-d-30830_irnt	T1D	-0.0009	0.0052	8.56E-01
Sitting height	ukb-a-195	T1D	0.0000	0.0049	9.92E-01
Sleep duration	ukb-a-9	T1D	-0.0208	0.0195	2.94E-01
Sleeplessness / insomnia	ukb-a-13	T1D	-0.0052	0.0154	7.39E-01
Sodium in urine	ukb-a-335	T1D	-0.0078	0.0293	7.93E-01
Standing height	ukb-a-389	T1D	-0.0022	0.0030	4.64E-01
Systolic blood pressure	ukb-a-360	T1D	0.0050	0.0103	6.28E-01
telomere length	ieu-b-4879	T1D	-0.0068	0.0063	2.85E-01
Testosterone	ukb-d-30850_irnt	T1D	-0.0018	0.0103	8.62E-01
Total bilirubin	ukb-d-30840_irnt	T1D	0.0003	0.0073	9.64E-01
Total cholesterol	ieu-a-301	T1D	-0.0031	0.0129	8.13E-01
Total protein	ukb-d-30860_irnt	T1D	0.0198	0.0091	3.07E-02
Townsend deprivation index at recruitment	ukb-a-44	T1D	-0.0316	0.0567	6.16E-01
Transferrin	ieu-a-1052	T1D	0.1620	0.2231	4.95E-01
Triglycerides	ukb-d-30870_irnt	T1D	0.0042	0.0045	3.48E-01
Trunk fat mass	ukb-a-291	T1D	-0.0090	0.0069	1.95E-01
Trunk fat percentage	ukb-a-290	T1D	0.0025	0.0094	7.89E-01
Trunk fat-free mass	ukb-a-292	T1D	-0.0047	0.0046	3.16E-01
Trunk predicted mass	ukb-a-293	T1D	-0.0042	0.0046	3.63E-01
Urate	ukb-d-30880_irnt	T1D	-0.0015	0.0038	6.98E-01
Urea	ukb-d-30670_irnt	T1D	-0.0117	0.0103	2.59E-01
Urinary sodium-potassium ratio	ieu-b-72	T1D	0.1657	0.1017	1.18E-01
Usual walking pace	ukb-a-513	T1D	-0.0295	0.0354	4.13E-01
Vitamin D	ukb-d-30890_irnt	T1D	0.0022	0.0061	7.21E-01
Waist circumference	ukb-a-382	T1D	-0.0032	0.0077	6.80E-01
Waist-to-hip ratio	ieu-a-72	T1D	0.0065	0.0318	8.40E-01
Weight	ukb-a-249	T1D	-0.0106	0.0054	5.05E-02
White blood cell (leukocyte) count	ukb-d-30000_irnt	T1D	0.0146	0.0074	4.94E-02
Whole body fat mass	ukb-a-265	T1D	-0.0088	0.0066	1.82E-01
Whole body fat-free mass	ukb-a-266	T1D	0.0000	0.0053	9.98E-01
Whole body water mass	ukb-a-267	T1D	0.0013	0.0068	8.47E-01
Adiponectin	ieu-a-1	T2D	-0.0136	0.0061	4.69E-02
Alanine aminotransferase	ukb-d-30620_irnt	T2D	0.0122	0.0040	2.91E-03
Albumin	ukb-d-30600_irnt	T2D	0.0112	0.0045	1.39E-02
Alcohol intake frequency	ukb-a-25	T2D	0.0461	0.0162	7.25E-03
Alcohol intake versus 10 years previously	ukb-a-32	T2D	0.1319	0.1322	3.64E-01
Alcoholic drinks per week	ieu-b-73	T2D	-0.0255	0.0108	2.47E-02
Alkaline phosphatase	ukb-d-30610_irnt	T2D	0.0014	0.0018	4.50E-01
Apoliprotein A	ukb-d-30630_irnt	T2D	-0.0063	0.0022	4.04E-03
Apoliprotein B	ukb-d-30640_irnt	T2D	0.0053	0.0027	4.97E-02
Aspartate aminotransferase	ukb-d-30650_irnt	T2D	0.0031	0.0024	2.00E-01
Basal metabolic rate	ukb-a-268	T2D	0.0104	0.0029	3.28E-04
Basophill percentage	ukb-d-30220_irnt	T2D	-0.0031	0.0028	2.63E-01
Birth weight	ukb-a-198	T2D	0.0312	0.0112	6.84E-03
Birth weight of first child	ukb-a-318	T2D	-0.0197	0.0163	2.34E-01

Body fat	ieu-a-999	T2D	-0.1395	0.0671	7.11E-02
Body fat percentage	ukb-a-264	T2D	-0.0091	0.0051	7.67E-02
body mass index	ieu-b-40	T2D	-0.0033	0.0022	1.29E-01
Calcium	ukb-d-30680_irnt	T2D	0.0054	0.0027	4.56E-02
Cholesterol	ukb-d-30690_irnt	T2D	0.0026	0.0031	4.08E-01
Cigarettes per Day	ieu-b-25	T2D	0.0085	0.0053	1.24E-01
C-reactive protein	ukb-d-30710_irnt	T2D	0.0028	0.0025	2.65E-01
Creatinine	ukb-d-30700_irnt	T2D	0.0002	0.0024	9.19E-01
Creatinine (enzymatic) in urine	ukb-a-333	T2D	0.0144	0.0180	4.35E-01
Current tobacco smoking	ukb-a-16	T2D	0.0259	0.0132	7.07E-02
Cystatin C	ukb-d-30720_irnt	T2D	0.0039	0.0016	1.70E-02
Daytime dozing / sleeping (narcolepsy)	ukb-a-15	T2D	-0.0248	0.0222	2.79E-01
Diastolic blood pressure	ukb-a-359	T2D	0.0021	0.0044	6.29E-01
Direct bilirubin	ukb-d-30660_irnt	T2D	-0.0044	0.0036	2.23E-01
Drive faster than motorway speed limit	ukb-a-8	T2D	-0.0274	0.0313	4.00E-01
Eosinophill percentage	ukb-d-30210_irnt	T2D	0.0012	0.0019	5.10E-01
Fasting glucose	ieu-b-114	T2D	0.0570	0.0190	5.55E-03
Fasting insulin	ieu-b-116	T2D	-0.0580	0.0331	1.05E-01
Fluid intelligence score	ukb-a-196	T2D	0.0052	0.0119	6.66E-01
Forced expiratory volume in 1-second (FEV1)	ukb-a-337	T2D	0.0097	0.0059	1.04E-01
Forced vital capacity (FVC)	ukb-a-336	T2D	0.0070	0.0040	8.08E-02
Gamma glutamyltransferase	ukb-d-30730_irnt	T2D	0.0071	0.0018	1.70E-04
Getting up in morning	ukb-a-10	T2D	0.0076	0.0163	6.42E-01
Glucose	ukb-d-30740_irnt	T2D	0.0259	0.0068	2.86E-04
Glycated haemoglobin	ukb-d-30750_irnt	T2D	0.0085	0.0034	1.23E-02
Haematocrit percentage	ukb-d-30030_irnt	T2D	0.0044	0.0024	7.11E-02
Haemoglobin concentration	ukb-d-30020_irnt	T2D	0.0042	0.0024	7.58E-02
HDL cholesterol	ukb-d-30760_irnt	T2D	-0.0105	0.0020	4.63E-07
Heart rate	ieu-a-1056	T2D	-0.0446	0.0142	8.67E-03
Heel bone mineral density (BMD) T-score	ukb-a-500	T2D	0.0011	0.0019	5.76E-01
High light scatter reticulocyte count	ukb-d-30300_irnt	T2D	0.0082	0.0024	6.73E-04
High light scatter reticulocyte percentage	ukb-d-30290_irnt	T2D	0.0076	0.0023	1.06E-03
Hip circumference	ukb-a-388	T2D	0.0038	0.0045	4.03E-01
IGF-1	ukb-d-30770_irnt	T2D	-0.0015	0.0020	4.40E-01
Immature reticulocyte fraction	ukb-d-30280_irnt	T2D	0.0036	0.0026	1.65E-01
Impedance of whole body	ukb-a-269	T2D	0.0031	0.0034	3.65E-01
Job involves mainly walking or standing	ukb-a-502	T2D	-0.0271	0.0545	6.39E-01
LDL direct	ukb-d-30780_irnt	T2D	0.0044	0.0027	9.97E-02
Length of menstrual cycle	ukb-a-351	T2D	0.0048	0.0139	7.46E-01
Lipoprotein A	ukb-d-30790_irnt	T2D	0.0059	0.0062	3.57E-01
Lymphocyte count	ukb-d-30120_irnt	T2D	0.0030	0.0021	1.40E-01
Lymphocyte percentage	ukb-d-30180_irnt	T2D	0.0003	0.0021	8.86E-01
Mean corpuscular haemoglobin	ukb-d-30050_irnt	T2D	-0.0031	0.0013	2.13E-02
Mean corpuscular haemoglobin concentration	ukb-d-30060_irnt	T2D	-0.0012	0.0034	7.34E-01
Mean corpuscular volume	ukb-d-30040_irnt	T2D	-0.0021	0.0015	1.60E-01

Mean platelet (thrombocyte) volume	ukb-d-30100_irnt	T2D	0.0012	0.0012	3.28E-01
Mean reticulocyte volume	ukb-d-30260_irnt	T2D	-0.0042	0.0019	3.26E-02
Mean sphered cell volume	ukb-d-30270_irnt	T2D	-0.0077	0.0020	1.45E-04
Mean time to correctly identify matches	ukb-a-199	T2D	0.0063	0.0179	7.26E-01
Microalbumin in urine	ukb-d-30500_irnt	T2D	0.0154	0.0282	6.40E-01
Monocyte count	ukb-d-30130_irnt	T2D	0.0018	0.0014	2.06E-01
Monocyte percentage	ukb-d-30190_irnt	T2D	0.0015	0.0016	3.59E-01
Morning/evening person (chronotype)	ukb-a-11	T2D	-0.0006	0.0052	9.15E-01
Nap during day	ukb-a-12	T2D	-0.0113	0.0076	1.45E-01
Neuroticism	ieu-a-1007	T2D	0.0255	0.0552	6.58E-01
Neuroticism score	ukb-a-230	T2D	0.0019	0.0125	8.82E-01
Neutrophill count	ukb-d-30140_irnt	T2D	0.0035	0.0024	1.46E-01
Neutrophill percentage	ukb-d-30200_irnt	T2D	-0.0009	0.0021	6.56E-01
Overall health rating	ukb-a-251	T2D	-0.0019	0.0112	8.65E-01
Past tobacco smoking	ukb-a-17	T2D	-0.0045	0.0101	6.54E-01
Peak expiratory flow (PEF)	ukb-a-338	T2D	0.0130	0.0112	2.49E-01
Phosphate	ukb-d-30810_irnt	T2D	0.0018	0.0027	5.11E-01
Platelet count	ukb-d-30080_irnt	T2D	-0.0004	0.0015	8.00E-01
Platelet crit	ukb-d-30090_irnt	T2D	-0.0025	0.0016	1.25E-01
Platelet distribution width	ukb-d-30110_irnt	T2D	0.0036	0.0014	9.13E-03
Potassium in urine	ukb-a-334	T2D	-0.0389	0.0399	3.62E-01
Pulse rate	ukb-a-3	T2D	0.0035	0.0029	2.34E-01
QRS duration	ukb-d-12340_irnt	T2D	-0.0031	0.0234	9.02E-01
Red blood cell (erythrocyte) count	ukb-d-30010_irnt	T2D	0.0037	0.0017	2.89E-02
Red blood cell (erythrocyte) distribution width	ukb-d-30070_irnt	T2D	0.0021	0.0019	2.78E-01
Reticulocyte count	ukb-d-30250_irnt	T2D	0.0088	0.0023	2.22E-04
Reticulocyte percentage	ukb-d-30240_irnt	T2D	0.0095	0.0025	1.78E-04
Serum cystatin C (eGFRcys)	ieu-a-1106	T2D	0.0074	0.0159	6.73E-01
SHBG	ukb-d-30830_irnt	T2D	-0.0115	0.0027	2.74E-05
Sitting height	ukb-a-195	T2D	0.0049	0.0023	3.20E-02
Sleep duration	ukb-a-9	T2D	-0.0054	0.0142	7.04E-01
Sleeplessness / insomnia	ukb-a-13	T2D	-0.0026	0.0098	7.97E-01
Sodium in urine	ukb-a-335	T2D	0.0184	0.0290	5.32E-01
Standing height	ukb-a-389	T2D	0.0009	0.0016	5.71E-01
Systolic blood pressure	ukb-a-360	T2D	0.0037	0.0041	3.71E-01
telomere length	ieu-b-4879	T2D	0.0016	0.0027	5.65E-01
Testosterone	ukb-d-30850_irnt	T2D	-0.0040	0.0041	3.30E-01
Total bilirubin	ukb-d-30840_irnt	T2D	-0.0059	0.0028	4.14E-02
Total cholesterol	ieu-a-301	T2D	0.0029	0.0034	3.90E-01
Total protein	ukb-d-30860_irnt	T2D	0.0059	0.0035	9.47E-02
Townsend deprivation index at recruitment	ukb-a-44	T2D	-0.0209	0.0178	3.24E-01
Transferrin	ieu-a-1052	T2D	-0.0107	0.0108	3.61E-01
Triglycerides	ukb-d-30870_irnt	T2D	0.0163	0.0028	2.30E-08
Trunk fat mass	ukb-a-291	T2D	0.0011	0.0045	8.13E-01
Trunk fat percentage	ukb-a-290	T2D	-0.0014	0.0055	8.07E-01

Trunk fat-free mass	ukb-a-292	T2D	0.0075	0.0027	5.54E-03
Trunk predicted mass	ukb-a-293	T2D	0.0075	0.0027	5.31E-03
Urate	ukb-d-30880_irnt	T2D	0.0027	0.0024	2.49E-01
Urea	ukb-d-30670_irnt	T2D	0.0054	0.0045	2.34E-01
Urinary sodium-potassium ratio	ieu-b-72	T2D	0.0496	0.0261	7.07E-02
Usual walking pace	ukb-a-513	T2D	-0.0176	0.0309	5.74E-01
Vitamin D	ukb-d-30890_irnt	T2D	-0.0029	0.0038	4.47E-01
Waist circumference	ukb-a-382	T2D	-0.0024	0.0044	5.84E-01
Waist-to-hip ratio	ieu-a-72	T2D	-0.0450	0.0248	8.03E-02
Weight	ukb-a-249	T2D	0.0048	0.0034	1.60E-01
White blood cell (leukocyte) count	ukb-d-30000_irnt	T2D	0.0026	0.0021	2.18E-01
Whole body fat mass	ukb-a-265	T2D	-0.0025	0.0042	5.54E-01
Whole body fat-free mass	ukb-a-266	T2D	0.0086	0.0028	1.99E-03
Whole body water mass	ukb-a-267	T2D	0.0078	0.0027	4.52E-03
Adiponectin	ieu-a-1	CKD	-0.0020	0.0085	8.18E-01
Alanine aminotransferase	ukb-d-30620_irnt	CKD	0.0019	0.0035	5.88E-01
Albumin	ukb-d-30600_irnt	CKD	-0.0008	0.0036	8.34E-01
Alcohol intake frequency	ukb-a-25	CKD	0.0116	0.0121	3.44E-01
Alcohol intake versus 10 years previously	ukb-a-32	CKD	0.0462	0.0829	6.01E-01
Alcoholic drinks per week	ieu-b-73	CKD	-0.0044	0.0141	7.58E-01
Alkaline phosphatase	ukb-d-30610_irnt	CKD	0.0019	0.0024	4.24E-01
Apoliprotein A	ukb-d-30630_irnt	CKD	-0.0090	0.0026	8.15E-04
Apoliprotein B	ukb-d-30640_irnt	CKD	0.0017	0.0029	5.54E-01
Aspartate aminotransferase	ukb-d-30650_irnt	CKD	0.0032	0.0031	2.96E-01
Basal metabolic rate	ukb-a-268	CKD	0.0025	0.0028	3.72E-01
Basophill percentage	ukb-d-30220_irnt	CKD	0.0013	0.0039	7.33E-01
Birth weight	ukb-a-198	CKD	0.0090	0.0091	3.27E-01
Birth weight of first child	ukb-a-318	CKD	-0.0308	0.0146	4.19E-02
Body fat	ieu-a-999	CKD	-0.0383	0.0492	4.58E-01
Body fat percentage	ukb-a-264	CKD	-0.0040	0.0053	4.53E-01
body mass index	ieu-b-40	CKD	-0.0007	0.0025	7.80E-01
Calcium	ukb-d-30680_irnt	CKD	0.0000	0.0032	9.89E-01
Cholesterol	ukb-d-30690_irnt	CKD	-0.0011	0.0036	7.71E-01
Cigarettes per Day	ieu-b-25	CKD	0.0132	0.0075	9.53E-02
C-reactive protein	ukb-d-30710_irnt	CKD	0.0033	0.0025	1.93E-01
Creatinine	ukb-d-30700_irnt	CKD	-0.0001	0.0038	9.80E-01
Creatinine (enzymatic) in urine	ukb-a-333	CKD	0.0425	0.0378	2.74E-01
Current tobacco smoking	ukb-a-16	CKD	0.0192	0.0219	3.96E-01
Cystatin C	ukb-d-30720_irnt	CKD	0.0063	0.0022	4.10E-03
Daytime dozing / sleeping (narcolepsy)	ukb-a-15	CKD	-0.0468	0.0211	4.03E-02
Diastolic blood pressure	ukb-a-359	CKD	0.0012	0.0078	8.79E-01
Direct bilirubin	ukb-d-30660_irnt	CKD	-0.0024	0.0036	5.13E-01
Drive faster than motorway speed limit	ukb-a-8	CKD	-0.0162	0.0292	5.90E-01
Eosinophill percentage	ukb-d-30210_irnt	CKD	-0.0030	0.0027	2.68E-01
Fasting glucose	ieu-b-114	CKD	0.0028	0.0120	8.14E-01

Fasting insulin	ieu-b-116	CKD	-0.0071	0.0273	7.99E-01
Fluid intelligence score	ukb-a-196	CKD	-0.0331	0.0163	4.95E-02
Forced expiratory volume in 1-second (FEV1)	ukb-a-337	CKD	0.0055	0.0059	3.47E-01
Forced vital capacity (FVC)	ukb-a-336	CKD	0.0057	0.0045	2.02E-01
Gamma glutamyltransferase	ukb-d-30730_irnt	CKD	-0.0015	0.0022	4.98E-01
Getting up in morning	ukb-a-10	CKD	-0.0153	0.0147	3.04E-01
Glucose	ukb-d-30740_irnt	CKD	0.0009	0.0048	8.44E-01
Glycated haemoglobin	ukb-d-30750_irnt	CKD	0.0057	0.0025	2.09E-02
Haematocrit percentage	ukb-d-30030_irnt	CKD	-0.0066	0.0032	3.65E-02
Haemoglobin concentration	ukb-d-30020_irnt	CKD	-0.0057	0.0029	5.02E-02
HDL cholesterol	ukb-d-30760_irnt	CKD	-0.0075	0.0023	9.62E-04
Heart rate	ieu-a-1056	CKD	-0.0360	0.0222	1.30E-01
Heel bone mineral density (BMD) T-score	ukb-a-500	CKD	0.0022	0.0028	4.29E-01
High light scatter reticulocyte count	ukb-d-30300_irnt	CKD	0.0011	0.0027	6.78E-01
High light scatter reticulocyte percentage	ukb-d-30290_irnt	CKD	0.0014	0.0027	5.92E-01
Hip circumference	ukb-a-388	CKD	-0.0042	0.0036	2.37E-01
IGF-1	ukb-d-30770_irnt	CKD	-0.0018	0.0023	4.35E-01
Immature reticulocyte fraction	ukb-d-30280_irnt	CKD	0.0068	0.0031	2.78E-02
Impedance of whole body	ukb-a-269	CKD	-0.0006	0.0033	8.49E-01
Job involves mainly walking or standing	ukb-a-502	CKD	-0.0104	0.0703	8.88E-01
LDL direct	ukb-d-30780_irnt	CKD	0.0026	0.0031	4.08E-01
Length of menstrual cycle	ukb-a-351	CKD	-0.0226	0.0162	2.37E-01
Lipoprotein A	ukb-d-30790_irnt	CKD	0.0157	0.0096	1.23E-01
Lymphocyte count	ukb-d-30120_irnt	CKD	0.0008	0.0030	7.88E-01
Lymphocyte percentage	ukb-d-30180_irnt	CKD	0.0039	0.0038	2.97E-01
Mean corpuscular haemoglobin	ukb-d-30050_irnt	CKD	-0.0018	0.0022	4.06E-01
Mean corpuscular haemoglobin concentration	ukb-d-30060_irnt	CKD	-0.0049	0.0054	3.65E-01
Mean corpuscular volume	ukb-d-30040_irnt	CKD	-0.0008	0.0022	7.19E-01
Mean platelet (thrombocyte) volume	ukb-d-30100_irnt	CKD	0.0010	0.0017	5.52E-01
Mean reticulocyte volume	ukb-d-30260_irnt	CKD	0.0022	0.0026	3.80E-01
Mean sphered cell volume	ukb-d-30270_irnt	CKD	0.0013	0.0024	5.76E-01
Mean time to correctly identify matches	ukb-a-199	CKD	0.0493	0.0301	1.14E-01
Microalbumin in urine	ukb-d-30500_irnt	CKD	-0.0245	0.0268	4.58E-01
Monocyte count	ukb-d-30130_irnt	CKD	-0.0016	0.0024	5.14E-01
Monocyte percentage	ukb-d-30190_irnt	CKD	0.0018	0.0022	4.21E-01
Morning/evening person (chronotype)	ukb-a-11	CKD	0.0040	0.0061	5.16E-01
Nap during day	ukb-a-12	CKD	-0.0084	0.0110	4.51E-01
Neuroticism	ieu-a-1007	CKD	0.1118	0.0495	5.83E-02
Neuroticism score	ukb-a-230	CKD	0.0183	0.0156	2.45E-01
Neutrophill count	ukb-d-30140_irnt	CKD	0.0032	0.0029	2.69E-01
Neutrophill percentage	ukb-d-30200_irnt	CKD	-0.0043	0.0031	1.63E-01
Overall health rating	ukb-a-251	CKD	-0.0055	0.0171	7.52E-01
Past tobacco smoking	ukb-a-17	CKD	-0.0143	0.0157	3.69E-01
Peak expiratory flow (PEF)	ukb-a-338	CKD	-0.0048	0.0091	5.96E-01
Phosphate	ukb-d-30810_irnt	CKD	0.0029	0.0029	3.27E-01

Platelet count	ukb-d-30080_irnt	CKD	-0.0015	0.0020	4.49E-01
Platelet crit	ukb-d-30090_irnt	CKD	-0.0070	0.0022	1.56E-03
Platelet distribution width	ukb-d-30110_irnt	CKD	0.0014	0.0018	4.57E-01
Potassium in urine	ukb-a-334	CKD	0.1226	0.0664	1.07E-01
Pulse rate	ukb-a-3	CKD	0.0050	0.0037	1.80E-01
QRS duration	ukb-d-12340_irnt	CKD	-0.0166	0.0387	6.97E-01
Red blood cell (erythrocyte) count	ukb-d-30010_irnt	CKD	0.0017	0.0026	5.10E-01
Red blood cell (erythrocyte) distribution width	ukb-d-30070_irnt	CKD	0.0000	0.0025	9.99E-01
Reticulocyte count	ukb-d-30250_irnt	CKD	0.0008	0.0026	7.48E-01
Reticulocyte percentage	ukb-d-30240_irnt	CKD	0.0025	0.0029	3.81E-01
Serum cystatin C (eGFRcys)	ieu-a-1106	CKD	-0.0418	0.0494	4.59E-01
SHBG	ukb-d-30830_irnt	CKD	-0.0037	0.0021	8.37E-02
Sitting height	ukb-a-195	CKD	-0.0015	0.0025	5.53E-01
Sleep duration	ukb-a-9	CKD	0.0145	0.0121	2.38E-01
Sleeplessness / insomnia	ukb-a-13	CKD	-0.0086	0.0114	4.58E-01
Sodium in urine	ukb-a-335	CKD	-0.0197	0.0161	2.32E-01
Standing height	ukb-a-389	CKD	-0.0009	0.0019	6.38E-01
Systolic blood pressure	ukb-a-360	CKD	-0.0037	0.0060	5.40E-01
telomere length	ieu-b-4879	CKD	0.0008	0.0040	8.47E-01
Testosterone	ukb-d-30850_irnt	CKD	-0.0046	0.0048	3.41E-01
Total bilirubin	ukb-d-30840_irnt	CKD	-0.0029	0.0027	2.85E-01
Total cholesterol	ieu-a-301	CKD	-0.0003	0.0041	9.46E-01
Total protein	ukb-d-30860_irnt	CKD	0.0061	0.0034	7.55E-02
Townsend deprivation index at recruitment	ukb-a-44	CKD	0.0655	0.0394	1.95E-01
Transferrin	ieu-a-1052	CKD	0.0140	0.0219	5.47E-01
Triglycerides	ukb-d-30870_irnt	CKD	0.0031	0.0023	1.80E-01
Trunk fat mass	ukb-a-291	CKD	-0.0019	0.0041	6.54E-01
Trunk fat percentage	ukb-a-290	CKD	0.0012	0.0057	8.40E-01
Trunk fat-free mass	ukb-a-292	CKD	-0.0002	0.0027	9.32E-01
Trunk predicted mass	ukb-a-293	CKD	-0.0006	0.0027	8.18E-01
Urate	ukb-d-30880_irnt	CKD	0.0044	0.0022	5.03E-02
Urea	ukb-d-30670_irnt	CKD	0.0028	0.0062	6.51E-01
Urinary sodium-potassium ratio	ieu-b-72	CKD	0.0164	0.0308	6.01E-01
Usual walking pace	ukb-a-513	CKD	-0.0189	0.0234	4.27E-01
Vitamin D	ukb-d-30890_irnt	CKD	-0.0037	0.0040	3.60E-01
Waist circumference	ukb-a-382	CKD	-0.0046	0.0044	3.04E-01
Waist-to-hip ratio	ieu-a-72	CKD	-0.0061	0.0214	7.79E-01
Weight	ukb-a-249	CKD	0.0004	0.0029	8.84E-01
White blood cell (leukocyte) count	ukb-d-30000_irnt	CKD	0.0030	0.0030	3.11E-01
Whole body fat mass	ukb-a-265	CKD	-0.0010	0.0038	7.97E-01
Whole body fat-free mass	ukb-a-266	CKD	0.0004	0.0027	8.80E-01
Whole body water mass	ukb-a-267	CKD	0.0007	0.0027	7.82E-01
